# The Effects of Mind-Body Interventions on Sleep Quality: A Systematic Review

**DOI:** 10.1155/2015/902708

**Published:** 2015-06-16

**Authors:** Rachel Neuendorf, Helané Wahbeh, Irina Chamine, Jun Yu, Kimberly Hutchison, Barry S. Oken

**Affiliations:** ^1^Helfgott Research Institute, National College of Natural Medicine, 049 SW Porter Street, Portland, OR 97201, USA; ^2^Oregon Health and Science University, 3181 SW Sam Jackson Park Road, CR-120, Portland, OR 97239, USA

## Abstract

*Study Objectives*. To evaluate the effect of mind-body interventions (MBI) on sleep. *Methods*. We reviewed randomized controlled MBI trials on adults (through 2013) with at least one sleep outcome measure. We searched eleven electronic databases and excluded studies on interventions not considering mind-body medicine. Studies were categorized by type of MBI, whether sleep was primary or secondary outcome measure and outcome type. *Results*. 1323 abstracts were screened, and 112 papers were included. Overall, 67 (60%) of studies reported a beneficial effect on at least one sleep outcome measure. Of the most common interventions, 13/23 studies using meditation, 21/30 using movement MBI, and 14/25 using relaxation reported at least some improvements in sleep. There were clear risks of bias for many studies reviewed, especially when sleep was not the main focus. *Conclusions*. MBI should be considered as a treatment option for patients with sleep disturbance. The benefit of MBI needs to be better documented with objective outcomes as well as the mechanism of benefit elucidated. There is some evidence that MBI have a positive benefit on sleep quality. Since sleep has a direct impact on many other health outcomes, future MBI trials should consider including sleep outcome measurements.

## 1. Introduction

Poor sleep quality is a common complaint in today's society [1]. About 25% of adults are not satisfied with their sleep, 10–15% have insomnia symptoms linked to negative daytime consequences, and 6–10% meet diagnostic criteria for insomnia [2]. Sleep-related disorders are often comorbid with medical or psychiatric disorders [3] and may increase the incidence of developing new onset psychiatric disorders such as depression, anxiety, and substance abuse [4, 5]. In addition, sleep problems may increase risk for cognitive impairment and dementia [6]. The consequences of sleep problems are far-reaching and result in significant burden for those affected, their families, and the work force [7].

An increasing number of adults are seeking treatment for sleep difficulties. The number of new generation hypnotics prescribed for sleep has increased by 430% over the past 11 years [8]. Despite many patients experiencing chronic symptoms, existing data support only short-term use of these medications [2]. Long-term use of hypnotic medications is associated with various adverse side effects ranging from dependence and tolerance to an increased risk for developing Alzheimer's disease [9]. As an alternative to pharmaceuticals, nonpharmacological interventions may provide safe and cost-effective treatment. There has been a growing interest in complementary and alternative medicine (CAM) approaches for optimizing health [10]. Mind-body interventions (MBI) are among the most commonly used CAM modalities [10]. The National Center for Complementary and Alternative Medicine defines MBI as the modalities focusing* on the interactions among the brain, mind, body, and behavior, with the intent to use the mind to affect physical functioning and promote health* [11]. About one in five adults in the US report using one or more MBI during the previous 12 months [12]. Among approximately 1.6 million US adults who use CAM therapies for sleep problems, MBI are among the most preferred [13]. MBI are increasingly incorporated into mainstream scientific research, and systematic reviews have indicated that mind-body approaches can improve common conditions such as depression [14] and chronic pain [15]. Since psychiatric and pain disorders frequently cooccur with sleep problems and may have bidirectional causal relationships [16], MBI may offer new promising treatments for chronic sleep disturbances and subsequent comorbidities. To date, numerous studies evaluating the efficacy of MBI for a variety of health conditions have included sleep measures as a part of their assessment; however, to our knowledge there have been no broad scope systematic reviews summarizing the effects of MBI on sleep among any diagnostic population. The overall goal of this review was to systematically evaluate the evidence of the effects of MBI on sleep. Our objectives were to (1) characterize the MBI studies where sleep outcomes were assessed, (2) evaluate the quality of these studies, and (3) assess the evidence associated with using MBI for sleep improvement.

## 2. Methods

### 2.1. Literature Search

We conducted comprehensive searches with the help of two research librarians with an end date of December 31, 2013, and varying start dates in MEDLINE (1950), Embase (1974), CINAHL (1982), PsycINFO (1967), AMED (1985), Alt HealthWatch (1984), Global Health (1973), Cochrane Central Register of Controlled Trials, Cochrane Database of Systematic Reviews (2005), and Database of Abstracts of Reviews of Effects. The search parameters for MBI, sleep outcomes, and subject types were kept broad and the search terms were customized for each database to optimize recall of the potential abstracts. When applicable, both the Medical Subject Headings (MeSH) terms and the keywords were searched. Terms relevant to MBI were first combined with sleep related terms, and various filters were then adopted according to the inclusion/exclusion criteria.

### 2.2. Study Eligibility

Two independent reviewers screened all of the abstracts according to the inclusion and exclusion criteria. The full text of the studies meeting the following inclusion criteria and those with insufficient information to determine eligibility from the abstract were retrieved. Only full-text articles that were published in English are included.

#### 2.2.1. Study Design

Randomized controlled trials (RCTs) with a sample size of 10 or more were included. All other study designs (nonrandomized, uncontrolled, observational, etc.) were excluded. All studies had to have an appropriate control that would allow for assessment of the impact of MBI. For example, studies that only compared two MBI to each other were excluded.

#### 2.2.2. Types of Participants

All adults (18 years or older) regardless of health conditions were included, except for shift workers and time zone travelers. Having a sleep complaint was not required.

#### 2.2.3. Interventions

All MBI as defined and classified in Wahbeh et al. [17] include biofeedback, guided imagery, hypnotherapy, meditation, relaxation, and movement MBI (yoga, qi gong, and tai chi). For this paper, sensory art therapies (aromatherapy, music therapy, etc.), spiritual therapies, psychoeducational interventions, breathing only interventions, and other CAM modalities that may incorporate a mind-body component (massage, acupuncture, etc.) were excluded. Trials studying MBI as a stand-alone intervention or as part of a multicomponent intervention were included, as long as they had an appropriate control that allowed for the mind-body intervention effect to be analyzed.

#### 2.2.4. Outcome Measures

Each study had to include at least one measurement of sleep. Subjective and objective sleep outcomes were assessed, including, but are not limited to, sleep quality, sleep duration, and sleep latency.

### 2.3. Data Extraction and Management

The following data were collected or computed: medical condition, mean age, whether sleep was primary or secondary outcome, gender, number of participants, mind-body intervention and delivery, home practice details (if any), time spent in each intervention, length of trial, time points at which outcomes were measured, control group, and subjective and objective sleep outcomes. Outcome *p* values were extracted for end of trial and any follow-up time points. A single reviewer extracted the data and another independent reviewer verified the accuracy and completeness of the extraction. Any discrepancies were resolved by consensus or a third party. All study data were managed with Microsoft Excel.

### 2.4. Assessment of Methodological Quality

Two reviewers independently evaluated the quality and risk of bias of each study in accordance with the* Cochrane Risk of Bias Tool* [18]. Any discrepancies were resolved by consensus or through a third party. The* Cochrane Risk of Bias Tool* evaluates selection, performance, detection, attrition, reporting, and other biases and is the current gold standard for assessing bias in RCTs. The criteria are categorized as high risk of bias, unclear risk of bias, or low risk of bias, and the categorization considers whether the risk of bias is sufficient enough to have a notable impact on the results or conclusions of the trial.

The overall quality of the paper was examined in two different ways. One way was with the Cochrane risk of bias (ROB) summary. With this method, a high risk in any category equates to a high ROB, an unclear risk in one or more categories without any high risk category equates to an unclear ROB, and all the categories need to be low risk to equate to a low ROB. Performance blinding category was excluded from the ROB assessment since most mind-body interventions are unable to have blinding. The second method of overall quality assessment was by evaluating the number of high risk and unclear risk in four of the categories (assessment, blinding, attrition, and reporting items). Papers with a score of 0 or 1 were considered low ROB studies.

### 2.5. Data Synthesis and Evidence Grading

Due to variation in participants, interventions, implementation, and outcomes across the studies, a meta-analysis or other evidence grading was not possible. Instead, our goal was to provide a general understanding of the current state of evidence for each intervention type. The studies were classified as positive (>25% of the measured sleep items had *p* value < 0.05 for between group over time comparison), mixed (>25% of the measured sleep items had *p* value < 0.05 in the mind-body intervention group over time or between mind-body intervention and control at postassessment, but no between group over time comparison reported), or negative (<25% of the measured items had *p* value < 0.05 favoring the mind-body intervention group compared to the control group). If the control condition was active the outcomes were classified as A and if nonactive they were classified as NA. Immediate versus sustained results were described separately. Sustained results were classified as any measurement taken one month or more after the end of intervention. Studies were grouped by intervention.

## 3. Results

### 3.1. Search Results

**Figure 1:**
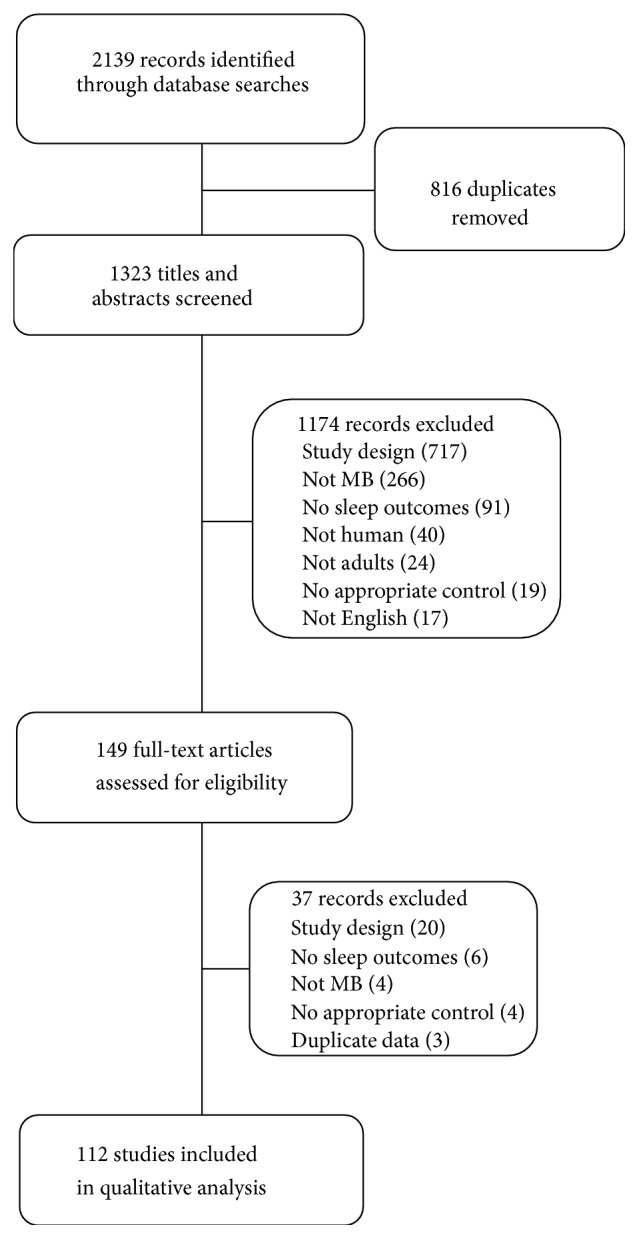
Study flow diagram.

A total of 2139 studies were identified (Figure 1). After removing duplicates, 1323 titles and abstracts were screened for inclusion criteria. 149 full-text articles were assessed for eligibility, and, of these, 112 were included in the final review (Table 1). Four of the included studies used multiple mind-body intervention groups and are listed in different categories.

### 3.2. Description of Included Studies

All manuscripts were published between 1975 and 2013. Sixty of the studies were conducted within the last 5 years (2009–2013). The mean sample size was 62 ± 51 (range 10–321). In total, 6830 participants were included (two studies did not report sample size). 36 studies used active control groups (either another intervention or placebo desensitization), 62 used nonactive controls (waitlist or treatment as usual), and 14 used active and nonactive controls. Fifty-five of the studies had sleep as the focus of the study with primary outcomes on sleep parameters, whereas 57 studies included sleep as secondary outcomes. Thirty-nine studies required sleep complaints as an inclusion criterion, whereas 73 did not. Participants were quite diverse, including elderly, women of menopausal, perimenopausal, and postmenopausal age, stressed working adults, medical and college students, veterans, inmates, cancer patients and survivors, people with insomnia, chronic pain, fibromyalgia, posttraumatic stress disorder, tinnitus, and Guillain-Barre syndrome, and patients undergoing hemodialysis and organ transplant. Four studies were all males, 27 studies were all females. The remaining studies had an average of 66% ± 19% females (range 6–97%). Five studies did not report participant gender. The following MBI were represented in the studies: biofeedback (12), guided imagery (3), hypnotherapy (11), meditation (23) (14 mindfulness meditation studies, 2 Transcendental Meditation studies, and 7 other types), mind-body movement (31) (3 qi gong studies, 20 yoga studies, 7 tai chi studies, and 1 Resseguier method), relaxation techniques (25) (1 Benson, 2, Applied, 1 Autogenic, 1 Home-based audio relaxation treatment, and 20 Progressive Muscle Relaxation). 11 studies investigated MBI as part of a multicomponent intervention. Subjective measures used to assess sleep varied and most studies used more than one instrument. The following self-reported assessment tools were used: individual sleep characteristics (e.g., total sleep time, sleep efficiency, sleep quality, sleep onset latency, quality of awakening, feeling refreshed upon waking, and sleep disturbance) (53), Pittsburgh Sleep Quality Index (34), Insomnia Severity Index (4), Medical Outcomes Study Sleep Scale (6), and nine other self-reported sleep outcome measures were used in only a single study. Objective measures used were actigraphy (3), polysomnography (10), and sleep spectrogram technique (1).

### 3.3. Assessment of Methodological Quality

Several of the studies failed to provide enough detail for adequate quality assessment. Methods of random sequence generation and allocation concealment were particularly poorly reported. Fifty-two studies had low risk on randomization, 53 had high risk, and 6 were rated as unclear risk. Fifty-five studies had low risk on concealment, 53 had high risk, and 4 were rated as unclear risk. Due to the nature of mind-body medicine interventions, almost all studies were unable to conduct performance blinding (110). Most studies had high (32) or unclear (57) risk for assessment blinding mostly because blinding of assessors or assessment procedures were not reported clearly or at all (low 23). Attrition risk was low in most studies (94) (high 6, unclear 12). Most studies had low risk for reporting (89) (high 7, unclear 16). Finally, most studies did not have other potential biases noted (99) (high 4, unclear 9).

### 3.4. Methodological Quality of Included Studies

Using the Cochrane ROB summary there were 12 studies with low ROB, 56 studies with unclear ROB, and 44 studies with high ROB. Using the numerical overall quality assessment method, there were 20 studies with a score of 0, 61 studies with a score of 1, 22 studies with a score of 2, and 9 studies with a score of 3. While in some cases a high ROB correlated with a score of 2 or more, there were instances where we ranked the study as good quality (0 or 1) but it was considered a high ROB with the Cochrane summary. For example, among the 20 yoga studies there were five studies where we scored a 0 or 1 but were considered a high ROB with Cochrane. There were also five studies with an unclear ROB where we scored a 0 or 1. The overall quality scores using the two methods are reported in Table 1.

### 3.5. Quality of the Body of Evidence for Each Modality

The body of evidence for each modality for sleep was reviewed for all included studies.

#### 3.5.1. Biofeedback

12 studies were included. For immediate effects, 8 were negative and 2 were mixed. Two of the studies used two control groups (active and nonactive). One study was positive for both controls and the other was positive for nonactive and negative for active control. All 3 studies that reported sustained effects were negative.

#### 3.5.2. Guided Imagery

Three studies were included. For immediate effects, 1 was mixed and 2 were negative. Only 1 study measured sustained effects and it was positive.

#### 3.5.3. Hypnotherapy

11 studies were included. For immediate effects, 5 were positive and 6 were negative. For the 4 studies that reported sustained effects, 1 was negative, 2 were positive, and 1 was mixed.

#### 3.5.4. Meditation

23 studies were included. For immediate effects, 8 were positive, 10 were negative, and 5 were mixed. For the 10 studies that reported sustained effects, 5 were positive and 5 were negative. Within the 23 total meditation studies, there were 14 mindfulness meditation studies that assessed immediate effects (positive (4), mixed (4), and negative (6)) and 6 that assessed sustained effects (positive (3) and negative (3)). There were two Transcendental Meditation studies and both were positive for immediate effects.

#### 3.5.5. Mind-Body Movement

31 studies were included. For immediate effects, 16 were positive, 9 were negative, and 3 were mixed. Three of the studies used both controls and two were positive for nonactive control but negative for active control and the other was negative for both controls. For the 7 studies that reported sustained effects, 1 was positive, 5 were negative, and 1 was mixed. Looking at the subcategories, there were 20 yoga studies that examined immediate effects (positive (10), mixed (1), and negative (8)) and one with two control groups (positive for nonactive and negative for active and the other was negative for both). All 4 yoga studies that report sustained effects were negative. There were three qi gong studies that examined immediate effects (positive (1), negative (1), and mixed (1)) and two that examined sustained effects (positive (1) and negative (1)). There were 7 studies with tai chi that examined immediate effects (positive (5), negative (1), and 1 with both controls (positive nonactive and negative active)).

#### 3.5.6. Relaxation Techniques

25 relaxation studies examined immediate effects (positive (5), negative (11), and mixed (4)). Five studies looked at both controls. Two were negative for active control/positive for nonactive, two were mixed for both, and one was positive for both. For sustained effects, there were 8 studies. Five studies were negative, 2 were positive, and one used both controls (negative for active and positive for nonactive).

#### 3.5.7. Multicomponent Studies

11 studies examined immediate effects (positive (5), negative (3), and mixed (3)). For sustained effects, there were 2 studies. One study was positive for both active and nonactive controls and one was negative.

## 4. Discussion

There were over one hundred RCTs using a mind-body therapy with at least one sleep outcome measure. The studies ranged in size from 10 to 321 participants. The study populations were quite variable, ranging from healthy people to patients with significant medical illnesses, for example, cancer. Less than half of the studies included participants with primary sleep complaints. While all studies were RCTs, the varied control groups consisted of passive controls (simple wait-list or treatment as usual controls) or active controls (attention and time matching or close matching in many characteristics, e.g., stretching for yoga or exercise for tai chi). While active controls are important to help determine the mechanism of action of any potential effect, they may be less critical to a clinician trying to make a clinical recommendation of a low risk, low cost treatment compared to a pharmacological treatment. Expectancy of improvement or placebo effects may be accounting for some of the positive findings; however, expectancy of improvement was rarely formally evaluated.

Overall quality of the studies was mixed, with only 13 studies achieving a low risk of bias from the Cochrane ROB. Of note, the Cochrane ROB measure is not well designed for nonparticipant blind trials (which is the case for almost all mind-body studies). Thus, we excluded participant blinding from the Cochrane ROB measure. The overall Cochrane ROB tool is not ideal for other reasons as well. It is limited to high, uncertain, and low ROB, causing lack of reporting certain information (e.g., details of blinding when the RCT appear to be of significant size and high quality) resulting in an uncertain rating. Additionally, the Cochrane ROB has its own limited reliability [132]. Given these issues with the Cochrane ROB tool, we developed a more stepwise scale that generally paralleled the Cochrane ROB. However, some studies with many uncertain ratings resulted in a poor quality rating and some well performed studies with just a single uncertain rating resulted in a reasonably high quality rating. Studies used both self-reported and objective sleep outcome measures although there were no obvious differences in study results between these two types of outcomes. There was some homogeneity on the subjective sleep measures although the diversity in intervention type and population precluded combining data from these studies. All these factors make it challenging to synthesize the results which are individually presented in Table 1 and Supplemental Table  1 in Supplementary Material available online at http://dx.doi.org/10.1155/2015/902708.

### 4.1. Biofeedback

The majority of the biofeedback studies were negative for sleep improvement compared to a control. The studies that showed positive or mixed results were low quality studies. The studies that measured sustained effects were all negative. Half of the studies focused on insomnia patients and required sleep complaints; seven studies included sleep measures as their primary outcome. Results from these studies were still mostly negative or mixed. No obvious differences were observed among studies of EEG biofeedback, EMG biofeedback, or other biofeedback modalities. At this point the evidence does not support the use of biofeedback for improvement in sleep. Inconclusive or negative findings of more specific reviews have similarly been reported. For example, a meta-analysis and systematic review of RCTs was conducted on the efficacy of EMG- and EEG-biofeedback in fibromyalgia syndrome with no evidence found for the reduction of sleep problems [133].

### 4.2. Guided Imagery

There were only three guided imagery studies included. The largest trial (86 participants) showed mixed results whereas the other 2 studies (total of 84 participants) were negative. Out of the three studies, only one focused on insomnia patients and measured sustained effects; the results were positive, indicating a potential long-term benefit of guided imagery among people with sleep complaints. There is insufficient evidence at this time regarding the use of guided imagery for sleep. Similarly, another review examining guided imagery for people with fibromyalgia was unable to calculate effect sizes for sleep benefits due to limited data available [134].

### 4.3. Hypnotherapy

The overall results for hypnotherapy were mixed. For immediate effects, four studies required sleep complaints and three reported positive effects against active controls. Two out of the three studies that recruited participants with insomnia were positive and one was negative. Two studies examining mental health conditions (PTSD (who also had insomnia) and mild depressive neurosis) were both positive. Two studies with fibromyalgia patients were negative for immediate results, but one of the studies showed positive sustained effect of hypnotherapy on sleep. Overall the results for the use of hypnotherapy for sleep are mixed. The current data suggest that condition selection may be an important component to the use of hypnotherapy. Future investigations assessing both immediate and sustained effects of hypnotherapy on sleep with special focus on insomnia or mental health patients are warranted.

### 4.4. Meditation

There were a substantial number of studies (23) assessing meditation effects, and diverse populations were involved. About a third of these studies reported positive effects of meditation on sleep. Half of the studies out of 10 assessing sustained effects of meditation were positive. Condition of the participant appears to be an important factor when recommending meditation or considering future research on meditation for sleep. On one hand, both studies conducted with veterans showed positive results. Two out of three studies focusing on patients in their middle age and older were also positive for immediate effects on sleep, and the remaining one was positive for sustained effects. On the other hand, three out of six studies conducted with patients with depression, stress, or anxiety were negative, and the rest had mixed results. Seven studies required sleep complaints and 11 studies included primary sleep outcomes, and most reported negative or mixed results; the two studies targeting patients with primary insomnia were both negative against active/pharmaceutical controls. No obvious differences were observed between results against active and nonactive controls. One previous systematic review examined Mindfulness-Based Stress Reduction on sleep disturbance found no clear positive effects of MBSR on sleep quality and duration; however, they did find evidence that increased practice was associated with improved sleep and decreased sleep-interfering cognitive processes [135]. Another large systematic review and meta-analysis of meditation programs found insufficient evidence of any effect on sleep, although there was trend favoring meditation [136]. Overall, the results for the use of meditation for sleep are mixed, and more research is needed to confirm benefits of using mediation in older populations and in veterans.

### 4.5. Mind-Body Movement

More than half of the 31 studies assessing mind-body movement were positive for immediate effects. Five studies required sleep complaints and 12 included sleep measures as primary outcomes. Almost all of these studies were positive. With ten studies indicating positive effect yoga had the highest amount of positive evidence. Three of these studies had the highest quality score both from Cochrane and from our summary score, and six studies had a score of 0 in our quality ranking (highest quality). Four of these positive yoga studies were conducted either in active cancer patients or cancer survivors. Eight studies assessing mind-body movement recruited elderly individuals, and seven of these studies (3 yoga studies, 4 tai chi studies) showed positive results. Similar findings were reported in a systematic review focused on yoga for the elderly [137]. Overall, there is some positive evidence to suggest benefit of mind-body movement therapies for sleep especially in certain populations (elderly and cancer patients/survivors). The majority of studies compared MBI against nonactive controls reported positive results, while those comparing against active controls yielded inconclusive evidence of benefits. Therefore, whether the effect of the therapies is due to the physical activity or the mind-body aspect is unclear as the majority of studies did not use physical activity as a control. Researchers planning future studies assessing mind-body movement modalities and sleep should consider using exercise control group in addition to other controls to help elucidate the mechanism underlying effects of mind-body movement modalities on sleep.

### 4.6. Relaxation Techniques

Of the 25 relaxation intervention studies, the majority were negative. Eighteen studies assessing relaxation were conducted with people suffering from sleep disturbances. Of these studies 4 reported positive results, 7 reported negative results, 2 reported mixed results, and 5 studies had both active and nonactive controls (for nonactive control: 3 positive and 2 mixed; for active control: 1 positive, 2 mixed, and 2 negative). In addition, eighteen studies included sleep measures as primary outcome and the results were fairly inconclusive. To date, the results are not conclusive but the data suggest that relaxation techniques may be useful for improved sleep in people with sleep problems. In a systematic review of nonpharmacologic interventions to improve sleep of hospitalized patients, relaxation therapies improved sleep quality 0–38%. However, similar to our experience, the authors state that “types and dose of interventions, outcome measures, length of follow-up, differences in patient populations, and dearth of randomized trials may dilute effects seen or make it more difficult to draw conclusions” [138].

### 4.7. Multicomponent Studies

Eleven of the reviewed studies included more than one mind-body intervention, and almost half of them showed positive results. The results suggest that there may be some benefit to combining different types of interventions to improve sleep but more systematic research on combining therapies is needed.

Overall, despite mixed and inconclusive results in this systematic review, several of the larger, high quality studies demonstrated effective mind-body therapies to improve sleep. Based on the reviewed data, the current evidence suggests that mind-body movement approaches, especially when used in the elderly populations, might be beneficial for sleep. The results of our review suggest that demographics and health condition may be important factors to consider when recommending mind-body modality to patients or when planning a research study.  Table 2 shows the results of the studies grouped by condition.

Mind-body therapies are low risk, making them attractive as an alternative to pharmacological therapy or as an addition to cognitive behavioral treatment. The ability of these treatments to foster states of relaxation, counteract intrusive thoughts, and decrease body tension is clearly in line with behaviors linked to improved sleep. MBI may be effective in sleep outcomes based on their effects on stress reduction [139]. Most mind-body therapies can be individually tailored and practiced at home, making treatments accessible to patients with a wide range of ages, comorbidities, and socioeconomic backgrounds.

There are limitations to this systematic review that need to be taken into account when considering its results. We chose to include all MBI and populations for this review to get a better understanding of what data currently exists for sleep outcomes. The broad scope makes it difficult to recommend treatment protocols for specific populations but does give an indication of where more research is needed. We chose to include any study that had a sleep outcome regardless of whether the participants had sleep complaints or whether the outcome was primary or secondary. This may have led to a flattening of effect because in studies where participants were not required to have a sleep complaint there would be a ceiling effect to how much their sleep outcomes could improve. Similarly, we did not take into account the mind-body intervention dose. For example, some studies had very brief dose of a couple of hours whereas others had much longer dose, for example, 6 months. The dose may play a pivotal role in the effects of the mind-body intervention. Unfortunately, because of the heterogeneity of participants, therapies, dose, and outcomes, meta-analysis or a more systematic grading of evidence was not possible. We were unable to utilize effect sizes in this review, since the studies had very different designs limiting the effect size interpretation. In addition, the effect sizes could not be calculated for some of the data that was available. Effect sizes that were examined ranged from no effect to large effect based on the usual descriptors. Finally, we only included studies published in English. Regardless, this review is the first of its kind to systematically organize and evaluate the data of MBI for sleep in a more global way and can be viewed as a starting point from which other reviews can build on.

## 5. Recommendations for Future Research

More high quality RCTs evaluating immediate and sustained effects of mind-body therapies on sleep are needed. More studies are needed in populations having primary sleep complaints. Further, the use of objective sleep assessments such as polysomnography or actigraphy in addition to subjective assessment of sleep quality is encouraged. Researchers should consider potential mechanisms that MBI may be working on with regard to improved sleep outcomes, such as chronic stress, and consider including those markers in the study. Higher quality studies with larger populations and appropriate blinding when possible are needed. We urge future researchers of MBI to consider sleep as a primary outcome, to maintain treatment for at least 4 weeks, and to maintain investigator blinding.

In summary, given the variable study populations and designs, the results are not conclusive. Some mind-body therapies studies produced mostly negative results, for example, biofeedback. Others such as meditation, hypnotherapy, and movement-based mind-body therapies (e.g., yoga) may produce some beneficial results, especially compared to wait-list or treatment as usual controls. Clinicians should consider this evidence and each patient's needs and interest when considering a mind-body intervention recommendation. Further research on mind-body interventions and sleep outcomes will support more conclusive results.

## Supplementary Material

Supplementary Table 1: includes the complete list of all included studies. The table includes the participant characteristics and a summary of the results used to assess the overall study outcome.

## Figures and Tables

**Table 1:** Summary of study quality and outcomes.

Study	*n*	Sleep 1° or 2°; sleep issue	ROB; quality score	Study outcome, immediate; sustained^*∗*^
Biofeedback				
Freedman and Papsdorf, 1976^*∗∗*^ [19]	18	1; Y	U; 0	Negative^A^; Negative^A^
Duivenvoorden and Van Dixhoorn, 1991 [20]	119	2; N	U; 1	Negative^A^; NFU
Haralambous et al., 1987 [21]	26	2; N	U; 1	Negative^NA^; NFU
Ebben et al., 2009 [22]	10	1; N	H; 1	Negative^NA^; NFU
Hauri, 1981 [23]	43	1; Y	H; 1	Negative^NA^; NFU
Lamontagne et al., 1975 [24]	23	2; N	H; 1	Negative^A^; NFU
Nicassio et al., 1982^*∗∗*^ [25]	22	1; Y	H; 2	Negative^A^; NFU
				Positive^NA^; NFU
Sanavio, 1988 [26]	24	1; Y	H; 2	Negative^A^; Negative^A^
VanderPlate and Eno, 1983 [27]	36	1; Y	H; 2	Positive^NA^; NFU
				Positive^A^; NFU
Levin, 1998 [28]	58	1; Y	U; 3	Mixed^A^; NFU
Yilmaz et al., 2010 [29]	39	2; N	U; 3	Mixed^A^; NFU
Lamontagne et al., 1977 [30]	75	2; N	H; 3	Negative^NA^; Negative^NA^

Guided imagery				
Casida et al., 2013 [31]	40	1; N	L; 0	Negative^NA^; NFU
Woolfolk and McNulty, 1983^*∗∗*^ [32]	44	1; Y	U; 1	Negative^NA^; Positive^NA^
Renzi et al., 2000 [33]	86	2; N	H; 1	Mixed^NA^; NFU

Hypnotherapy				
Carnahan et al., 2010 [34]	38	1; Y	U; 1	Negative^NA^; NFU
Picard et al., 2013 [35]	59	2; N	U; 1	Negative^NA^; Positive^NA^
Castel et al., 2012 [36]	93	2; N	H; 1	Negative^A^; Negative^A^
Elkins et al., 2013 [37]	174	2; N	H; 1	Positive^A^; Positive^A^
Abrahamsen et al., 2008 [38]	41	2; N	H; 2	Negative^A^; NFU
Abrahamsen et al., 2009 [39]	40	2; N	H; 2	Negative^A^; NFU
Abramowitz et al., 2008 [40]	32	2; Y	H; 2	Positive^A^; Mixed^A^
Elkins et al., 2008 [41]	51	2; N	H; 2	Positive^NA^; NFU
Barabasz, 1976 [42]	NR	1; Y	U; 3	Positive^A^; NFU
Whitehouse et al., 1996 [43]	35	2; N	U; 3	Negative^NA^; NFU
Stanton, 1989 [44]	45	1; Y	H; 3	Positive^A^; NFU

Meditation: mindfulness				
Britton et al., 2010 [45]	21	1; Y	L; 0	Negative^NA^; NFU
Britton et al., 2012 [46]	24	1; Y	L; 0	Negative^NA^; NFU
Esmer et al., 2010 [47]	25	2; N	U; 1	Positive^NA^; Positive^NA^
Gross et al., 2011 [48]	27	1; Y	U; 1	Negative^A^; Negative^A^
Stötter et al., 2013 [49]	28	2; N	U; 1	Mixed^NA^; NFU
Andersen et al., 2013 [50]	247	1; N	H; 1	Mixed^NA^; Negative^NA^
Carmody et al., 2011 [51]	92	2; N	H; 1	Positive^NA^; Negative^NA^
Gross et al., 2010 [52]	122	1; N	H; 1	Positive^A^; Positive^A^
Lengacher et al., 2012 [53]	84	2; N	H; 1	Negative^NA^; NFU
Nakamura et al., 2013 [54]	35	1; Y	H; 1	Mixed^A^; Positive^A^
Wolever et al., 2012^*∗∗*^ [55]	205	2; N	H; 1	Positive^NA^; NFU
Klatt et al., 2009 [56]	45	2; N	U; 2	Mixed^NA^; NFU
Malarkey et al., 2013 [57]	170	2; N	H; 2	Negative^A^; NFU
Shapiro et al., 2003 [58]	54	1; N	H; 3	Negative^A^; NFU

Meditation: other				
Chan et al., 2012 [59]	51	1; Y	U; 0	Mixed^A^; NFU
				Mixed^NA^; NFU
Milbury et al., 2013 [60]	40	2; N	U; 1	Negative^NA^; Negative^NA^
Nakamura et al., 2011 [61]	58	1; Y	U; 1	Positive^A^; NFU
Pinniger et al., 2013 [62]	64	1; N	U; 1	Negative^NA^; Negative^NA^
Rybarczyk et al., 1999 [63] + Rybarczyk et al., 2001 [64]	237	2; N	U; 1	Positive^NA^; Positive^NA^
Schoicket et al., 1988 [65]	65	1; Y	U; 1	Negative^A^; NFU
Wiriyasombat et al., 2011 [66]	77	1; N	U; 1	Negative^NA^; Positive^NA^

Meditation: TM				
Travis et al., 2009 [67]	38	2; N	L; 0	Positive^NA^
Brooks and Scarano, 1985 [68]	18	2; N	U; 1	Positive^A^

Movement: yoga				
Innes and Selfe, 2012 [69]	20	1; N	L; 0	Positive^A^; NFU
Mustian et al., 2013 [70]	321	1; Y	L; 0	Positive^NA^; NFU
Yurtkuran et al., 2007 [71]	37	2; N	L; 0	Positive^NA^; NFU
Afonso et al., 2012 [72]	44	1; Y	U; 0	Positive^NA^; NFU
				Negative^A^; NFU
Hariprasad et al., 2013 [73]	87	1; N	U; 0	Positive^NA^; NFU
Vadiraja et al., 2009 [74]	75	2; N	H; 0	Positive^A^; NFU
Chattha et al., 2008 [75]	108	2; N	U; 1	Negative^A^; NFU
Cohen et al., 2004 [76]	38	1; N	U; 1	Positive^NA^; NFU
Köhn et al., 2013 [77]	37	2; N	U; 1	Negative^NA^; NFU
Bower et al., 2012 [78]	31	2; N	H; 1	Negative^A^; Negative^A^
Chen et al., 2010 [79]	55	1; N	H; 1	Positive^NA^; NFU
Elavsky and McAuley, 2007 [80]	150	1; Y	H; 1	Negative^A^; NFU
				Negative^NA^; NFU
Wolever et al., 2012^*∗∗*^ [55]	205	2; N	H; 1	Positive^NA^; NFU
Manjunath and Telles, 2005 [81]	55	1; N	U; 2	Negative^NA^; Negative^NA^
Carson et al., 2009 [82]	29	2; N	H; 2	Positive^NA^; Negative^NA^
Chandwani et al., 2010 [83]	56	2; N	H; 2	Negative^NA^; Negative^NA^
Chen et al., 2009 [84]	128	1; N	H; 2	Positive^NA^; NFU
Garfinkel et al., 1998 [85]	42	2; N	H; 2	Negative^NA^; NFU
Sakuma et al., 2012 [86]	82	2; N	H; 2	Negative^NA^; NFU
Dhruva et al., 2012 [87]	16	2; N	H; 3	Mixed^NA^; NFU

Movement: qi qong				
Liu et al., 2012 [88]	12	2; N	U; 1	Mixed^A^; NFU
Lynch et al., 2012 [89]	73	2; N	U; 1	Positive^NA^; Positive^NA^
Chen et al., 2013 [90]	96	2; N	H; 1	Negative^NA^; Negative^NA^

Movement: Resseguier				
Bongi et al., 2010 [91]	41	2; N	L; 0	Mixed^NA^; Mixed^NA^

Movement: tai chi				
Jones et al., 2012 [92]	98	2; N	L; 0	Positive^A^; NFU
Wang et al., 2010 [93]	29	2; N	U; 0	Negative^A^; NFU
Nguyen and Kruse, 2012 [94]	73	1; N	U; 1	Positive^NA^; NFU
Yeh et al., 2008 [95]	18	1; N	U; 1	Positive^NA^; NFU
Irwin et al., 2008 [96]	112	1; Y	H; 1	Positive^A^; NFU
Frye et al., 2007 [97]	72	2; N	U; 2	Negative^A^; NFU
				Positive^NA^; NFU
Li et al., 2004 [98]	118	1; Y	H; 2	Positive^A^; NFU

Multiple MB				
Sendhilkumar et al., 2013 [99]	20	2; N	L; 0	Positive^NA^; NFU
Carlson et al., 2001 [100]	44	2; N	U; 1	Mixed^NA^; Negative^NA^
Chen and Francis, 2010 [101]	15	2; N	U; 1	Mixed^NA^; NFU
Cohen and Fried, 2007 [102]	114	2; N	U; 1	Positive^A^; Positive^A^
				Positive^NA^; Positive^NA^
Field et al., 2013 [103]	75	2; N	U; 1	Positive^NA^; NFU
Richardson, 2003 [104]	36	1; N	U; 1	Negative^NA^; NFU
Yang et al., 2010 [105]	79	2; N	U; 1	Positive^A^; NFU
				Positive^NA^; NFU
Richards, 1998 [106]	70	1; N	H; 1	Negative^NA^; NFU
				Negative^A^; NFU
Sun et al., 2013 [107]	75	1; Y	H; 1	Positive^A^; NFU
Toussaint et al., 2012 [108]	21	2; N	H; 1	Negative^NA^; NFU
Sumter et al., 2009 [109]	33	2; N	U; 2	Mixed^NA^; NFU

Relaxation: other				
Gustavsson and von Koch, 2006 [110]	33	2; N	L; 0	Negative^NA^; Negative^NA^
Giblin and Clift, 1983 [111]	20	1; Y	U; 1	Negative^NA^; NFU
Lindh-Åstrand and Nedstrand, 2013 [112]	58	2; N	U; 1	Positive^NA^; Positive^NA^
Rambod et al., 2013 [113]	83	1; N	U; 1	Mixed^NA^; NFU
Rybarczyk et al., 2002 [114]	41	1; Y	U; 1	Negative^A^; Negative^A^
				Positive^NA^; Positive^NA^

Relaxation: PMR				
Edinger et al., 2001 [115]	70	1; Y	L; 0	Negative^A^; NFU
Francis and D'Silva, 2012 [116]	60	1; Y	U; 0	Mixed^NA^; NFU
Freedman and Papsdorf, 1976^*∗∗*^ [19]	18	1; Y	U; 0	Negative^A^; Negative^A^
Borkovec and Weerts, 1976 [117]	33	1; Y	H; 0	Mixed^A^; Positive^A^
				Mixed^NA^; NFU
Cannici et al., 1983 [118]	30	1; Y	U; 1	Negative^NA^; NFU
Engle-Friedman et al., 1992 [119]	NR	1; Y	U; 1	Negative^A^; NFU
				Positive^NA^; NFU
Espie et al., 1989 [120]	70	1; Y	U; 1	Positive^A^; Negative^A^
Field et al., 1999 [121]	26	2; N	U; 1	Negative^A^; NFU
Ducloux et al., 2013 [122]	18	1; Y	H; 2	Negative^NA^; Negative^NA^
Bae et al., 2012 [123]	24	2; N	H; 3	Mixed^NA^; NFU
Ireland et al., 1985 [124]	28	2; N	U; 1	Negative^NA^; NFU
Lick and Heffler, 1977 [125]	40	1; Y	U; 1	Positive^A^; NFU
				Positive^NA^; NFU
Wang et al., 2012 [126]	130	2; Y	U; 1	Mixed^NA^; NFU
				Mixed^A^; NFU
Wilson, 1982 [127]	64	2; N	U; 1	Negative^A^; NFU
Woolfolk and McNulty, 1983^*∗∗*^ [32]	44	1; Y	U; 1	Negative^NA^; Negative^NA^
Lacks et al., 1983 [128]	64	1; Y	H; 1	Negative^A^; NFU
Lichstein et al., 1999 [129]	30	1; Y	U; 2	Positive^NA^; NFU
Waters et al., 2003 [130]	53	1; Y	U; 2	Mixed^NA^; NFU
Greeff and Conradie, 1998 [131]	22	1; Y	H; 2	Positive^NA^; NFU
Nicassio et al., 1982^*∗∗*^ [25]	22	1; Y	H; 2	Positive^NA^; NFU

^*∗*^Studies were classified as positive (>25% of the measured sleep items had *p* value < 0.05 for between group over time comparison), mixed (>25% of the measured sleep items had *p* value < 0.05 in the mind-body intervention group over time or between mind-body intervention and control at postassessment, but no between group over time comparison reported), or negative (<25% of the measured items had *p* value < 0.05 favoring the mind-body intervention group compared to the control group).

^*∗∗*^Studies used multiple MBI and are, therefore, listed in different categories.

A = active control comparison; NA = nonactive control; NFU = no follow-up; H = high risk of bias, U = unclear risk of bias, and L = low risk of bias.

**Table 2:** Mind-body intervention study findings for specific diseases or conditions.

Diagnoses, symptoms, conditions	Modalities assessed	Positive findings	Mixed findings	Negative findings
Atopic dermatitis	PMR		Bae, 2012^NA,H/3^	

Cancer and cancer-related symptoms	Hypnosis			Carnahan, 2010^NA,U/1^
	MM	Nakamura, 2013^A,H/1, *∗*^	Andersen, 2013^NA,H/1^ Nakamura, 2013^A,H/1^	Andersen, 2013^NA,H/1,*∗*^ Lengacher, 2012^NA,H/1^ Shapiro, 2003^A,H/3^
	M other			Milbury, 2013^NA,U/1^
	Multiple	Cohen, 2007^A/NA,U/1^ Yang, 2010^A/NA,U/1^		
	PMR			Cannici, 1983^NA,U/1^ Ducloux, 2013^NA,H/2^
	Qi qong			Chen, 2013^NA,H/1^
	Yoga	Carson, 2009^NA,H/2^ Cohen, 2004^A,U/1^ Mustian, 2013^NA,L/0^ Vadiraja, 2009^A,H/0^	Dhruva, 2012^NA,H/3^	Bower, 2012^NA,H/1^ Carson, 2009^NA,H/2,*∗*^ Chandwani, 2010^NA,H/2^

Cardiovascular disease	Biofeedback			Duivenvoorden, 1991^A,U/1^
	GI			Casida, 2013^NA,L/0^
	Multiple			Richards, 1998^A/NA,H/1^
	Tai chi	Yeh, 2008^NA,U/1^		

Carpal syndrome	Yoga			Garfinkel, 1998^A,H/2^

Chronic health condition	Biofeedback		Yilmaz, 2010^A,U/3^	
	M other	Rybarczuk, 1999, 2001^NA,U/1^		

Chronic pain conditions	Hypnosis	Picard, 2013^NA,U/1,*∗*^		Abrahamsen, 2008^A,H/2^ Abrahamsen, 2009^A,H/2^ Castel, 2012^A,H/1^ Picard, 2013^NA,U/1^
	Multiple		Carlson, 2001^NA,U/1^ Chen, 2010^NA,U/1^	Carlson, 2001^NA,U/1,*∗*^ Toussaint, 2012^NA,H/1^
	Qi qong	Lynch, 2012^NA,U/1^	Liu, 2012^A,U/1^	
	Relaxation			Gustavsson, 2006^NA,L/0^
	Resseguier		Bongi, 2010^NA,L/0^	
	Tai chi	Jones, 2012^A,L/0^		

Critically ill	Multiple			Richardson, 2003^ NA,U/1^

Depression and depressive disorders	Hypnosis	Barabasz, 1976^A,U/3^		
	MM		Stotter, 2013^NA,U/1^	Britton, 2010^NA,L/0^ Britton, 2012^NA,L/0^
	M other		Chan, 2012^A/NA,U/0^	Pinniger, 2013^A/NA,U/1^
	Multiple	Field, 2013^NA,U/1^		
	PMR			Wilson 1982^A,U/1^

Elderly	M other	Wiriyasombat, 2011^NA,L/0,*∗*^		Wiriyasombat, 2011^NA,L/0^
	Multiple	Sun, 2013^A,H/1^		
	Tai chi	Frye, 2007^NA,U/2^ Irwin, 2008^A,H/1^ Li, 2004^A,H/2^ Nguyen, 2012^NA,U/1^		Frye, 2007^A,U/2^ Wang, 2010^A,U/0^
	Yoga	Chen, 2009^NA,H/2^ Chen, 2010^NA,H/1^ Hariprasad, 2013^NA,U/0^		Manjunath, 2005^NA,U/2^

Guillain-Barre	Multiple	Sendhilkumar, 2013^NA,L/0^		

Healthy volunteers	Biofeedback			Ebben, 2009^NA,H/1^, Lamontagne, 1975^A,H/1^ Lamontagne, 1977^A/NA,H/3^
	Hypnosis			Whitehouse, 1996^NA,U/3^
	MM	Wolever, 2012^NA,H/1^		Malarkey, 2013^A,H/1^
	Multiple		Sumter, 2009^NA,U/2^	
	TM	Travis, 2009^NA,L/0^		
	Yoga	Innes, 2012^A,L/0^ Wolever, 2012^NA,H/1^		Sakuma, 2012^NA,H/2^

Hospital admission	PMR		Francis, 2012^NA,U/0^	

Insomnia and sleep disorders	Biofeedback	Nicassio, 1982^NA,H/2^ VanderPlate, 1983^A/NA,H/2^	Levin, 1998^A,U/3^	Freedman, 1976^A,U/0^ Hauri, 1981^NA,H/1^ Nicassio, 1982^A,H/2^ Sanavio, 1988^A,H/2^
	GI	Woolfolk, 1983^NA,U/1,*∗*^		Woolfolk, 1983^NA,U/1^
	Hypnosis	Stanton, 1989^A,H/3^		
	MM			Gross, 2011^A,U/1^
	M other			Schoicket, 1988^A,U/1^
	PMR	Borkovec, 1976^A,H/0,*∗*^ Engle-Friedman, 1992^NA,U/1^ Espie, 1989^A,U/1^ Greeff, 1998^NA,H/2^ Lichstein, 1999^NA,U/2^ Lick, 1977^A/NA,U/1^ Nicassio, 1982^NA,H/2^	Borkovec, 1976^A/NA,H/0^ Wang, 2012^A/NA,U/1^ Waters, 2003^NA,U/2^	Edinger, 2001^A,L/0^ Engle-Friedman, 1992^A,U/1^ Espie, 1989^A,U/1,*∗*^ Freedman, 1976^A,U/0^ Lacks, 1983^A,H/1^ Woolfolk, 1983^NA,U/1^
	Relaxation	Rybarczyk, 2002^NA,U/1^		Giblin, 1983^NA,U/1^ Rybarczyk, 2002^A,U/1^
	Yoga	Afonso, 2012^NA,U/0^		Afonso, 2012^A,U/0^

Perimenopause and menopause	Yoga			Chattha, 2008^A,U/1^ Elavsky, 2007^A/NA,H/1^

Postmenopausal hot flashes	Hypnosis	Elkins, 2013^A,H/1^ Elkins, 2008^NA,H/2^		
	MM	Carmody, 2011^A,H/1^		Carmody, 2011^A,H/1,*∗*^
	Relaxation	Lindh-Åstrand, 2013^NA,U/1^		

Pregnancy	PMR			Field, 1999^A,U/1^

PTSD or veterans with insomnia symptoms	Hypnosis	Abramowitz, 2008^A,H/2^		
	M other	Nakamura, 2011^A,U/1^		
	TM	Brooks, 1985^A,U/1^		

Stress-related disorders	Yoga		Klatt, 2009^NA,U/2^	Köhn, 2013^NA,U/1^

Surgeries/invasive procedures	GI		Renzi, 2000^NA,H/1^	
	MM	Esmer, 2010^NA,U/1^ Gross, 2010^A,H/1^		
	Relaxation		Rambod, 2013^NA,U/1^	
	Yoga	Yurtkuran, 2007^NA,L/0^		

Tinnitus	Biofeedback			Haralambous, 1987^NA,U/1^
	PMR			Ireland, 1985^NA,U/1^

GI: guided imagery, MM: mindfulness mediation, M other: other type of mediation, Multiple: studies assessing multiple mind body modalities, PMR: progressive muscle relaxation, TM: transcendental meditation.

Superscript indices

A: active control and NA: nonactive control.

L: low risk of bias rating (Cochrane), U: unknown risk of bias rating (Cochrane), and H: high risk of bias rating (Cochrane).

0–3: quality scores provided by the authors with lowest score reflecting lowest risk of bias.

*∗*: reflecting only sustained outcome results if different from the immediate outcome results.

## References

[1] Michal M., Wiltink J., Kirschner Y. (2014). Complaints of sleep disturbances are associated with cardiovascular disease: results from the gutenberg health study. *PLoS ONE*.

[2] Morin C. M., Benca R. (2012). Chronic insomnia. *The Lancet*.

[3] Budhiraja R., Roth T., Hudgel D. W., Budhiraja P., Drake C. L. (2011). Prevalence and polysomnographic correlates of insomnia comorbid with medical disorders. *Sleep*.

[4] Chang P. P., Ford D. E., Mead L. A., Cooper-Patrick L., Klag M. J. (1997). Insomnia in young men and subsequent depression. The Johns Hopkins Precursors Study. *American Journal of Epidemiology*.

[5] Breslau N., Roth T., Rosenthal L., Andreski P. (1996). Sleep disturbance and psychiatric disorders: a longitudinal epidemiological study of young adults. *Biological Psychiatry*.

[6] Yaffe K., Laffan A. M., Harrison S. L. (2011). Sleep-disordered breathing, hypoxia, and risk of mild cognitive impairment and dementia in older women. *The Journal of the American Medical Association*.

[7] National Institutes of Health (2005). NIH State-of-the-Science Conference Statement on manifestations and management of chronic insomnia in adults. *NIH Consensus and State-of-the-Science Statements*.

[8] Ford E. S., Wheaton A. G., Cunningham T. J., Giles W. H., Chapman D. P., Croft J. B. (2014). Trends in outpatient visits for insomnia, sleep apnea, and prescriptions for sleep medications among US adults: findings from the national ambulatory medical care survey 1999–2010. *Sleep Medicine Reviews*.

[9] Billioti de Gage S., Morid Y., Ducruet T. (2014). Benzodiazepine use and risk of Alzheimer’s disease: case-control study. *BMJ Case Reports*.

[10] Barnes P. M., Bloom B., Nahin R. L. (2008). Complementary and alternative medicine use among adults and children: United States, 2007. *National Health Statistics Reports*.

[11] National Center for Complementary and Alternative Medicine http://nccam.nih.gov/video/series/mindbody.

[12] Wolsko P. M., Eisenberg D. M., Davis R. B., Phillips R. S. (2004). Use of mind-body medical therapies. *Journal of General Internal Medicine*.

[13] Pearson N. J., Johnson L. L., Nahin R. L. (2006). Insomnia, trouble sleeping, and complementary and alternative medicine: analysis of the 2002 national health interview survey data. *Archives of Internal Medicine*.

[14] D'Silva S., Poscablo C., Habousha R., Kogan M., Kligler B. (2012). Mind-body medicine therapies for a range of depression severity: a systematic review. *Psychosomatics*.

[15] Sierpina V., Astin J., Giordano J. (2007). Mind-body therapies for headache. *American Family Physician*.

[16] Nowell P. D., Buysse D. J. (2001). Treatment of insomnia in patients with mood disorders. *Depress Anxiety*.

[17] Wahbeh H., Elsas S. M., Oken B. S. (2008). Mind-body interventions: applications in neurology. *Neurology*.

[18] Higgins J. P., Altman D. G., Gotzsche P. C. (2011). The Cochrane Collaboration's tool for assessing risk of bias in randomised trials. *The British Medical Journal*.

[19] Freedman R., Papsdorf J. D. (1976). Biofeedback and progressive relaxation treatment of sleep-onset insomnia—a controlled, all-night investigation. *Biofeedback and Self-Regulation*.

[20] Duivenvoorden H. J., Van Dixhoorn J. (1991). Predictability of psychic outcome for exercise training and exercise training including relaxation therapy after myocardial infarction. *Journal of Psychosomatic Research*.

[21] Haralambous G., Wilson P. H., Platt-Hepworth S., Tonkin J. P., Hensley V. R., Kavanagh D. (1987). EMG biofeedback in the treatment of tinnitus: an experimental evaluation. *Behaviour Research and Therapy*.

[22] Ebben M. R., Kurbatov V., Pollak C. P. (2009). Moderating laboratory adaptation with the use of a heart-rate variability biofeedback device (StressEraser). *Applied Psychophysiology & Biofeedback*.

[23] Hauri P. (1981). Treating psychophysiologic insomnia with biofeedback. *Archives of General Psychiatry*.

[24] Lamontagne Y., Hand I., Annable L., Gagnon M. A. (1975). Physiological and psychological effects of alpha and EMG feedback training with college drug users: a pilot study. *Canadian Psychiatric Association Journal*.

[25] Nicassio P. M., Boylan M. B., McCabe T. G. (1982). Progressive relaxation, EMG biofeedback and biofeedback placebo in the treatment of sleep-onset insomnia. *The British Journal of Medical Psychology*.

[26] Sanavio E. (1988). Pre-sleep cognitive intrusions and treatment of onset-insomnia. *Behaviour Research and Therapy*.

[27] VanderPlate C., Eno E. N. (1983). Electromyograph biofeedback and sleep onset insomnia: comparison of treatment and placebo. *Behavioral Engineering*.

[28] Levin Y. I. (1998). ‘Brain music’ in the treatment of patients with insomnia. *Neuroscience and Behavioral Physiology*.

[29] Yilmaz O. O., Senocak O., Sahin E. (2010). Efficacy of EMG-biofeedback in knee osteoarthritis. *Rheumatology International*.

[30] Lamontagne Y., Beausejour R., Annable L., Tetreault T. (1977). Alpha and EMG feedback training in the prevention of drug abuse. A controlled study. *Canadian Psychiatric Association Journal*.

[31] Casida J. M., Yaremchuk K. L., Shpakoff L., Marrocco A., Babicz G., Yarandi H. (2013). The effects of guided imagery on sleep and inflammatory response in cardiac surgery: a pilot randomized controlled trial. *Journal of Cardiovascular Surgery*.

[32] Woolfolk R. L., McNulty T. F. (1983). Relaxation treatment for insomnia: a component analysis. *Journal of Consulting & Clinical Psychology*.

[33] Renzi C., Peticca L., Pescatori M. (2000). The use of relaxation techniques in the perioperative management of proctological patients: preliminary results. *International Journal of Colorectal Disease*.

[34] Carnahan L. F., Ritterband L. M., Bailey E. T., Thorndike F. P., Lord H. R., Baum L. D. (2010). Results from a study examining the feasibility and preliminary efficacy of a self-hypnosis intervention available on the web for cancer survivors with insomnia. *E-Journal of Applied Psychology*.

[35] Picard P., Jusseaume C., Boutet M., Dualé C., Mulliez A., Aublet-Cuvellier B. (2013). Hypnosis for management of fibromyalgia. *International Journal of Clinical and Experimental Hypnosis*.

[36] Castel A., Cascón R., Padrol A., Sala J., Rull M. (2012). Multicomponent cognitive-behavioral group therapy with hypnosis for the treatment of fibromyalgia: Long-Term outcome. *Journal of Pain*.

[37] Elkins G. R., Fisher W. I., Johnson A. K., Carpenter J. S., Keith T. Z. (2013). Clinical hypnosis in the treatment of postmenopausal hot flashes: a randomized controlled trial. *Menopause*.

[38] Abrahamsen R., Baad-Hansen L., Svensson P. (2008). Hypnosis in the management of persistent idiopathic orofacial pain—clinical and psychosocial findings. *Pain*.

[39] Abrahamsen R., Zachariae R., Svensson P. (2009). Effect of hypnosis on oral function and psychological factors in temporomandibular disorders patients. *Journal of Oral Rehabilitation*.

[40] Abramowitz E. G., Barak Y., Ben-Avi I., Knobler H. Y. (2008). Hypnotherapy in the treatment of chronic combat-related PTSD patients suffering from insomnia: A randomized, zolpidem-controlled clinical trial. *International Journal of Clinical and Experimental Hypnosis*.

[41] Elkins G., Marcus J., Stearns V. (2008). Randomized trial of a hypnosis intervention for treatment of hot flashes among breast cancer survivors. *Journal of Clinical Oncology*.

[42] Barabasz A. F. (1976). Treatment of insomnia in depressed patients by hypnosis and cerebral electrotherapy. *The American Journal of Clinical Hypnosis*.

[43] Whitehouse W. G., Dinges D. F., Orne E. C. (1996). Psychosocial and immune effects of self-hypnosis training for stress management throughout the first semester of medical school. *Psychosomatic Medicine*.

[44] Stanton H. E. (1989). Hypnotic relaxation and the reduction of sleep onset insomnia. *International Journal of Psychosomatics*.

[45] Britton W. B., Haynes P. L., Fridel K. W., Bootzin R. R. (2010). Polysomnographic and subjective profiles of sleep continuity before and after mindfulness-based cognitive therapy in partially remitted depression. *Psychosomatic Medicine*.

[46] Britton W. B., Haynes P. L., Fridel K. W., Bootzin R. R. (2012). Mindfulness-based cognitive therapy improves polysomnographic and subjective sleep profiles in antidepressant users with sleep complaints. *Psychotherapy & Psychosomatics*.

[47] Esmer G., Blum J., Rulf J., Pier J. (2010). Mindfulness-based stress reduction for failed back surgery syndrome: a randomized controlled trial. *Journal of the American Osteopathic Association*.

[48] Gross C. R., Kreitzer M. J., Reilly-Spong M. (2011). Mindfulness-based stress reduction versus pharmacotherapy for chronic primary insomnia: a randomized controlled clinical trial. *Explore: The Journal of Science and Healing*.

[49] Stötter A., Mitsche M., Endler P. C. (2013). Mindfulness-based touch therapy and mindfulness practice in persons with moderate depression. *Body, Movement and Dance in Psychotherapy*.

[50] Andersen S. R., Würtzen H., Steding-Jessen M. (2013). Effect of mindfulness-based stress reduction on sleep quality: results of a randomized trial among Danish breast cancer patients. *Acta Oncologica*.

[51] Carmody J. F., Crawford S., Salmoirago-Blotcher E., Leung K., Churchill L., Olendzki N. (2011). Mindfulness training for coping with hot flashes: results of a randomized trial. *Menopause*.

[52] Gross C. R., Kreitzer M. J., Thomas W. (2010). Mindfulness-based stress reduction for solid organ transplant recipients: a randomized controlled trial. *Alternative Therapies in Health & Medicine*.

[53] Lengacher C. A., Reich R. R., Post-White J. (2012). Mindfulness based stress reduction in post-treatment breast cancer patients: an examination of symptoms and symptom clusters. *Journal of Behavioral Medicine*.

[54] Nakamura Y., Lipschitz D. L., Kuhn R., Kinney A. Y., Donaldson G. W. (2013). Investigating efficacy of two brief mind-body intervention programs for managing sleep disturbance in cancer survivors: a pilot randomized controlled trial. *Journal of Cancer Survivorship*.

[55] Wolever R. Q., Bobinet K. J., McCabe K. (2012). Effective and viable mind-body stress reduction in the workplace: a randomized controlled trial. *Journal of Occupational Health Psychology*.

[56] Klatt M. D., Buckworth J., Malarkey W. B. (2009). Effects of low-dose mindfulness-based stress reduction (MBSR-ld) on working adults. *Health Education & Behavior*.

[57] Malarkey W. B., Jarjoura D., Klatt M. (2013). Workplace based mindfulness practice and inflammation: a randomized trial. *Brain, Behavior, and Immunity*.

[58] Shapiro S. L., Bootzin R. R., Figueredo A. J., Lopez A. M., Schwartz G. E. (2003). The efficacy of mindfulness-based stress reduction in the treatment of sleep disturbance in women with breast cancer. An exploratory study. *Journal of Psychosomatic Research*.

[59] Chan A. S., Wong Q. Y., Sze S. L., Kwong P. P. K., Han Y. M. Y., Cheung M.-C. (2012). A Chinese Chan -based mind-body intervention improves sleep on patients with depression: a randomized controlled trial. *The Scientific World Journal*.

[60] Milbury K., Chaoul A., Biegler K. (2013). Tibetan sound meditation for cognitive dysfunction: results of a randomized controlled pilot trial. *Psycho-Oncology*.

[61] Nakamura Y., Lipschitz D. L., Landward R., Kuhn R., West G. (2011). Two sessions of sleep-focused mind-body bridging improve self-reported symptoms of sleep and PTSD in veterans: a pilot randomized controlled trial. *Journal of Psychosomatic Research*.

[62] Pinniger R., Thorsteinsson E. B., Brown R. F., McKinley P. (2013). Tango dance can reduce distress and insomnia in people with self-referred affective symptoms. *The American Journal of Dance Therapy*.

[63] Rybarczyk B., DeMarco G., DeLaCruz M., Lapidos S. (1999). Comparing mind-body wellness interventions for older adults with chronic illness: classroom versus home instruction. *Behavioral Medicine*.

[64] Rybarczyk B., DeMarco G., DeLaCruz M., Lapidos S., Fortner B. (2001). A classroom mind/body wellness intervention for older adults with chronic illness: comparing immediate and 1-year benefits. *Behavioral Medicine*.

[65] Schoicket S. L., Bertelson A. D., Lacks P. (1988). Is sleep hygiene a sufficient treatment for sleep-maintenance insomnia?. *Behavior Therapy*.

[66] Wiriyasombat R., Pothiban L., Panuthai S., Sucamvang K., Saengthong S. (2011). Effectiveness of Buddhist doctrine practice-based programs in enhancing spiritual well-being, coping and sleep quality of Thai elders. *Pacific Rim International Journal of Nursing Research*.

[67] Travis F., Haaga D. A. F., Hagelin J. (2009). Effects of Transcendental Meditation practice on brain functioning and stress reactivity in college students. *International Journal of Psychophysiology*.

[68] Brooks J. S., Scarano T. (1985). Transcendental Meditation in the treatment of post-Vietnam adjustment. *Journal of Counseling & Development*.

[69] Innes K. E., Selfe T. K. (2012). The effects of a gentle yoga program on sleep, mood, and blood pressure in older women with restless legs syndrome (RLS): a preliminary randomized controlled trial. *Evidence-based Complementary & Alternative Medicine*.

[70] Mustian K. M., Sprod L. K., Janelsins M. (2013). Multicenter, randomized controlled trial of yoga for sleep quality among cancer survivors. *Journal of Clinical Oncology*.

[71] Yurtkuran M., Alp A., Dilek K. (2007). A modified yoga-based exercise program in hemodialysis patients: a randomized controlled study. *Complementary Therapies in Medicine*.

[72] Afonso R. F., Hachul H., Kozasa E. H. (2012). Yoga decreases insomnia in postmenopausal women: a randomized clinical trial. *Menopause*.

[73] Hariprasad V. R., Sivakumar P. T., Koparde V. (2013). Effects of yoga intervention on sleep and quality-of-life in elderly: a randomized controlled trial. *Indian Journal of Psychiatry*.

[74] Vadiraja H. S., Rao R. M., Hongasandra N. R. (2009). Effects of yoga on symptom management in breast cancer patients: a randomized controlled trial. *International Journal of Yoga*.

[75] Chattha R., Nagarathna R., Padmalatha V., Nagendra H. R. (2008). Effect of yoga on cognitive functions in climacteric syndrome: a randomised control study. *BJOG: An International Journal of Obstetrics & Gynaecology*.

[76] Cohen L., Warneke C., Fouladi R. T., Rodriguez M. A., Chaoul-Reich A. (2004). Psychological adjustment and sleep quality in a randomized trial of the effects of a Tibetan yoga intervention in patients with lymphoma. *Cancer*.

[77] Köhn M., Lundholm U. P., Bryngelsson I.-L., Anderzén-Carlsson A., Westerdahl E. (2013). Medical yoga for patients with stress-related symptoms and diagnoses in primary health care: a randomized controlled trial. *Evidence-Based Complementary and Alternative Medicine*.

[78] Bower J. E., Garet D., Sternlieb B. (2012). Yoga for persistent fatigue in breast cancer survivors: a randomized controlled trial. *Cancer*.

[79] Chen K.-M., Chen M.-H., Lin M.-H., Fan J.-T., Lin H.-S., Li C.-H. (2010). Effects of yoga on sleep quality and depression in elders in assisted living facilities. *Journal of Nursing Research*.

[80] Elavsky S., McAuley E. (2007). Lack of perceived sleep improvement after 4-month structured exercise programs. *Menopause*.

[81] Manjunath N. K., Telles S. (2005). Influence of Yoga & Ayurveda on self-rated sleep in a geriatic population. *Indian Journal of Medical Research*.

[82] Carson J. W., Carson K. M., Porter L. S., Keefe F. J., Seewaldt V. L. (2009). Yoga of Awareness program for menopausal symptoms in breast cancer survivors: results from a randomized trial. *Supportive Care in Cancer*.

[83] Chandwani K. D., Thornton B., Perkins G. H. (2010). Yoga improves quality of life and benefit finding in women undergoing radiotherapy for breast cancer.

[84] Chen K.-M., Chen M.-H., Chao H.-C., Hung H.-M., Lin H.-S., Li C.-H. (2009). Sleep quality, depression state, and health status of older adults after silver yoga exercises: cluster randomized trial. *International journal of nursing studies*.

[85] Garfinkel M. S., Singhal A., Katz W. A., Allan D. A., Reshetar R., Schumacher H. R. (1998). Yoga-based intervention for carpal tunnel syndrome: a randomized trial. *Journal of the American Medical Association*.

[86] Sakuma Y., Sasaki-Otomaru A., Ishida S. (2012). Effect of a home-based simple yoga program in child-care workers: a randomized controlled trial. *Journal of Alternative & Complementary Medicine*.

[87] Dhruva A., Miaskowski C., Abrams D. (2012). Yoga breathing for cancer chemotherapy-associated symptoms and quality of life: results of a pilot randomized controlled trial. *Journal of Alternative and Complementary Medicine*.

[88] Liu W., Zahner L., Cornell M. (2012). Benefit of Qigong exercise in patients with fibromyalgia: a pilot study. *International Journal of Neuroscience*.

[89] Lynch M., Sawynok J., Hiew C., Marcon D. (2012). A randomized controlled trial of qigong for fibromyalgia. *Arthritis Research and Therapy*.

[90] Chen Z., Meng Z., Milbury K. (2013). Qigong improves quality of life in women undergoing radiotherapy for breast cancer: results of a randomized controlled trial. *Cancer*.

[91] Bongi S. M., Di Felice C., Del Rosso A. (2010). The efficacy of the Rességuier method in the treatment of fibromyalgia syndrome: a randomized controlled trial. *Clinical & Experimental Rheumatology*.

[92] Jones K. D., Sherman C. A., Mist S. D., Carson J. W., Bennett R. M., Li F. (2012). A randomized controlled trial of 8-form Tai chi improves symptoms and functional mobility in fibromyalgia patients. *Clinical Rheumatology*.

[93] Wang W., Sawada M., Noriyama Y. (2010). Tai Chi exercise versus rehabilitation for the elderly with cerebral vascular disorder: a single-blinded randomized controlled trial. *Psychogeriatrics*.

[94] Nguyen M. H., Kruse A. (2012). A randomized controlled trial of Tai chi for balance, sleep quality and cognitive performance in elderly Vietnamese. *Clinical Interventions in Aging*.

[95] Yeh G. Y., Mietus J. E., Peng C.-K. (2008). Enhancement of sleep stability with Tai Chi exercise in chronic heart failure: preliminary findings using an ECG-based spectrogram method. *Sleep Medicine*.

[96] Irwin M. R., Olmstead R., Motivala S. J. (2008). Improving sleep quality in older adults with moderate sleep complaints: a randomized controlled trial of Tai Chi Chih. *Sleep*.

[97] Frye B., Scheinthal S., Kemarskaya T., Pruchno R. (2007). Tai chi and low impact exercise: effects on the physical functioning and psychological well-being of older people. *Journal of Applied Gerontology*.

[98] Li F., Fisher K. J., Harmer P., Irbe D., Tearse R. G., Weimer C. (2004). Tai chi and self-rated quality of sleep and daytime sleepiness in older adults: a randomized controlled trial. *Journal of the American Geriatrics Society*.

[99] Sendhilkumar R., Gupta A., Nagarathna R., Taly A. B. (2013). Effect of pranayama and meditation as an add-on therapy in rehabilitation of patients with Guillain-Barré syndrome—a randomized control pilot study. *Disability and Rehabilitation*.

[100] Carlson C. R., Bertrand P. M., Dale Ehrlich A., Maxwell A. W., Burton R. G. (2001). Physical self-regulation training for the management of temporomandibular disorders. *Journal of Orofacial Pain*.

[101] Chen Y. L., Francis A. J. P. (2010). Relaxation and imagery for chronic, nonmalignant pain: effects on pain symptoms, quality of life, and mental health. *Pain Management Nursing*.

[102] Cohen M., Fried G. (2007). Comparing relaxation training and cognitive-behavioral group therapy for women with breast cancer. *Research on Social Work Practice*.

[103] Field T., Diego M., Delgado J., Medina L. (2013). Tai chi/yoga reduces prenatal depression, anxiety and sleep disturbances. *Complementary Therapies in Clinical Practice*.

[104] Richardson S. (2003). Effects of relaxation and imagery on the sleep of critically ill adults. *Dimensions of Critical Care Nursing*.

[105] Yang X.-L., Li H.-H., Hong M.-H., Kao H. S. R. (2010). The effects of Chinese calligraphy handwriting and relaxation training in Chinese Nasopharyngeal Carcinoma patients: a randomized controlled trial. *International Journal of Nursing Studies*.

[106] Richards K. C. (1998). Effect of a back massage and relaxation intervention on sleep in critically ill patients. *The American Journal of Critical Care*.

[107] Sun J., Kang J., Wang P., Zeng H. (2013). Self-relaxation training can improve sleep quality and cognitive functions in the older: a one-year randomised controlled trial. *Journal of Clinical Nursing*.

[108] Toussaint L. L., Whipple M. O., Abboud L. L., Vincent A., Wahner-Roedler D. L. (2012). A mind-body technique for symptoms related to fibromyalgia and chronic fatigue. *Explore: The Journal of Science and Healing*.

[109] Sumter M. T., Monk-Turner E., Turner C. (2009). The benefits of meditation practice in the correctional setting. *Journal of Correctional Health Care*.

[110] Gustavsson C., von Koch L. (2006). Applied relaxation in the treatment of long-lasting neck pain: a randomized controlled pilot study. *Journal of Rehabilitation Medicine*.

[111] Giblin M. J., Clift A. D. (1983). Sleep without drugs. *Journal of the Royal College of General Practitioners*.

[112] Lindh-Åstrand L., Nedstrand E. (2013). Effects of applied relaxation on vasomotor symptoms in postmenopausal women: a randomized controlled trial. *Menopause*.

[113] Rambod M., Pourali-Mohammadi N., Pasyar N., Rafii F., Sharif F. (2013). The effect of Benson's relaxation technique on the quality of sleep of Iranian hemodialysis patients: a randomized trial. *Complementary Therapies in Medicine*.

[114] Rybarczyk B., Lopez M., Benson R., Alsten C., Stepanski E. (2002). Efficacy of two behavioral treatment programs for comorbid geriatric insomnia. *Psychology & Aging*.

[115] Edinger J. D., Wohlgemuth W. K., Radtke R. A., Marsh G. R., Quillian R. E. (2001). Cognitive behavioral therapy for treatment of chronic primary insomnia a randomized controlled trial. *The Journal of the American Medical Association*.

[116] Francis N., D'Silva F. (2012). Effectiveness of progressive muscle relaxation therapy on quality of sleep among patients admitted in medical ward of a selected hospital in Mangalore. *International Journal of Nursing Education*.

[117] Borkovec T. D., Weerts T. C. (1976). Effects of progressive relaxation on sleep disturbance: an electroencephalographic evaluation. *Psychosomatic Medicine*.

[118] Cannici J., Malcolm R., Peek L. A. (1983). Treatment of insomnia in cancer patients using muscle relaxation training. *Journal of Behavior Therapy & Experimental Psychiatry*.

[119] Engle-Friedman M., Bootzin R. R., Hazlewood L., Tsao C. (1992). An evaluation of behavioral treatments for insomnia in the older adult. *Journal of Clinical Psychology*.

[120] Espie C. A., Lindsay W. R., Brooks D. N., Hood E. M., Turvey T. (1989). A controlled comparative investigation of psychological treatments for chronic sleep-onset insomnia. *Behaviour Research & Therapy*.

[121] Field T., Hernandez-Reif M., Hart S., Theakston H., Schanberg S., Kuhn C. (1999). Pregnant women benefit from massage therapy. *Journal of Psychosomatic Obstetrics and Gynaecology*.

[122] Ducloux D., Guisado H., Pautex S. (2013). Promoting sleep for hospitalized patients with advanced cancer with relaxation therapy: experience of a randomized study. *American Journal of Hospice & Palliative Medicine*.

[123] Bae B. G., Oh S. H., Park C. O. (2012). Progressive muscle relaxation therapy for atopic dermatitis: objective assessment of efficacy. *Acta Dermato-Venereologica*.

[124] Ireland C. E., Wilson P. H., Tonkin J. P., Platt-Hepworth S. (1985). An evaluation of relaxation training in the treatment of tinnitus. *Behaviour Research and Therapy*.

[125] Lick J. R., Heffler D. (1977). Relaxation training and attention placebo in the treatment of severe insomnia. *Journal of Consulting and Clinical Psychology*.

[126] Wang W., He G., Wang M., Liu L., Tang H. (2012). Effects of patient education and progressive muscle relaxation alone or combined on adherence to continuous positive airway pressure treatment in obstructive sleep apnea patients. *Sleep & Breathing*.

[127] Wilson P. H. (1982). Combined pharmacological and behavioural treatment of depression. *Behaviour Research and Therapy*.

[128] Lacks P., Bertelson A. D., Gans L., Kunkel J. (1983). The effectiveness of three behavioral treatments for different degrees of sleep onset insomnia. *Behavior Therapy*.

[129] Lichstein K. L., Peterson B. A., Riedel B. W., Means M. K., Epperson M. T., Aguillard R. N. (1999). Relaxation to assist sleep medication withdrawal. *Behavior Modification*.

[130] Waters W. F., Hurry M. J., Binks P. G. (2003). Behavioral and hypnotic treatments for insomnia subtypes. *Behavioral Sleep Medicine*.

[131] Greeff A. P., Conradie W. S. (1998). Use of progressive relaxation training for chronic alcoholics with insomnia. *Psychological Reports*.

[132] Armijo-Olivo S., Ospina M., da Costa B. R. (2014). Poor reliability between Cochrane reviewers and blinded external reviewers when applying the Cochrane risk of bias tool in physical therapy trials. *PLoS ONE*.

[133] Glombiewski J. A., Bernardy K., Hauser W. (2013). Efficacy of EMG- and EEG-biofeedback in fibromyalgia syndrome: a meta-analysis and a systematic review of randomized controlled trials. *Evidence-Based Complementary and Alternative Medicine*.

[134] Bernardy K., Fuber N., Klose P., Hauser W. (2011). Efficacy of hypnosis/guided imagery in fibromyalgia syndrome—a systematic review and meta-analysis of controlled trials. *BMC Musculoskeletal Disorders*.

[135] Winbush N. Y., Gross C. R., Kreitzer M. J. (2007). The effects of mindfulness-based stress reduction on sleep disturbance: a systematic review. *Explore*.

[136] Goyal M., Singh S., Sibinga E. M. (2014). Meditation programs for psychological stress and well-being: a systematic review and meta-analysis. *JAMA Internal Medicine*.

[137] Wang Y. Y., Chang H. Y., Lin C. Y. (2014). Systematic review of yoga for depression and quality of sleep in the elderly. *Hu Li Za Zhi (Journal of Nursing)*.

[138] Tamrat R., Huynh-Le M. P., Goyal M. (2014). Non-pharmacologic interventions to improve the sleep of hospitalized patients: a systematic review. *Journal of General Internal Medicine*.

[139] Oken B. S., Chamine I., Wakeland W. (2015). A systems approach to stress, stressors and resilience in humans. *Behavioural Brain Research*.

