# Supplementary material for: The Effects of Mind-Body Interventions on Sleep Quality: A Systematic Review
Source: Evid Based Complement Alternat Med. 2015 Jun 16;2015:902708. doi: 10.1155/2015/902708 (PMC4487927; doi:10.1155/2015/902708)
Supplement: Supplementary file 1 — Supplementary Table 1: includes the complete list of all included studies. The table includes the participant characteristics and a summary of the results used to assess the overall study outcome. [file 902708.f1.pdf]

Supplemental Table 1: Summary of results for all included studies.

| Study | n | Mean age yrs ± SD [Range]; % female | Participant characteristics | Sleep 1° or 2° outcome; Sleep complaint required | Intervention vs comparator | Outcome Measurements | Study Outcome Immediate* | Study Outcome sustained* | Results |
|---|---|---|---|---|---|---|---|---|---|
| BIOFEEDBACK | | | | | | | | | |
| Duivenvoorden 1991 | 119 | NR | Cardiac patients after myocardial infarction | 2<br>N | EMG Frontalis Biofeedback vs. PE | SRS | Negative[A] | N/A | No sig. difference between groups on self-reported sleep quality over the 6 weeks. |
| Ebben 2009 | 10 | 20; 70% | Healthy | 1<br>N | HRV Biofeedback vs. NTC | PSG, PSQI | Negative[NA] | N/A | No sig. difference in any of the outcomes between groups except with stage 1 sleep (p<0.05) and sleep disturbance scores (p=0.003) |
| Freedman 1976* | 18 | [17-39]; 56% | Insomnia | 1<br>Y | Biofeedback vs Williams exercises | PSG | Negative[A] | Negative[A] | Sig. improvement in 1/5 of the PSG measurements reported at 2 weeks but not at 2 month follow-up. |
| Haralambous 1987 | 26 | 50.9 [25-76]; 50% | Tinnitus | 2<br>N | EMG Biofeedback vs. WL | SRS | Negative[NA] | N/A | No sig. improvement between groups. |
| Hauri 1981 | 43 | 41.3 ± 14.6; 63% | Insomnia | 1<br>Y | EMG Biofeedback vs. NTC | PSG, SD | Negative[NA] | N/A | No sig. improvement with intervention in any objective sleep measurements. All groups compared together. |
| | | | | | SMR Biofeedback vs. NTC | | Negative[NA] | N/A | |
| Lamontagne 1975 | 23 | 19; 29% | Cannabis user; college students | 2<br>N | EEG alpha vs. Sham Biofeedback | SRS | Negative[A] | N/A | No sig. difference in sleep outcomes compared to active sham NFB control. Both groups improved. |
| | | | | | EMG vs. Sham Biofeedback | | Negative[A] | N/A | No sig. difference in sleep outcomes compared to active sham NFB control. Both groups improved. |

| | | | | | | | | | |
|---|---|---|---|---|---|---|---|---|---|
| Lamontagne 1977 | 75 | 20.5 [17-25]; NR | Cannabis user; college students | 2 N | EEG alpha vs. Sham Biofeedback | SRS | Negative[A] | Negative[A] | No sig. improvement between groups in immediate or sustained effects. |
| | | | | | EEG alpha vs. NTC | | Negative[NA] | Negative[NA] | |
| | | | | | EMG vs. Sham Biofeedback | | Negative[A] | Negative[A] | |
| | | | | | EMG vs. NTC | | Negative[NA] | Negative[NA] | |
| Levin 1998 | 58 | 43 [18-60]; 60% | Insomnia | 1 Y | Brain music Biofeedback vs. Placebo brain music | PSG, SD, SRS | Mixed[A] | N/A | Sig. improvement in pre-post sleep measurements with intervention and not control (p<0.05). No between group comparisons given. |
| Nicassio 1982* | 22 | 43.5 [17-64]; 78% | Insomnia | 1 Y | EMG biofeedback vs. Biofeedback placebo | SRS, SD | Negative[A] | N/A | No sig. difference between biofeedback and placebo. |
| | | | | | EMG biofeedback vs. NTC | | Positive[NA] | N/A | Sig. improvement in sleep outcomes with biofeedback compared to control (p<0.05). |
| Sanavio 1988 | 24 | 39 [23-59]; 58% | Insomnia | 1 Y | Biofeedback vs. CBT | SD | Negative[A] | Negative[A] | No sig. improvement between groups. |
| VanderPlate 1983 | 36 | 20; 100% | Insomnia; undergraduates | 1 Y | EMG Biofeedback vs. Self-monitoring | SBQ, SD | Positive[NA] | N/A | Sig. improvement in 5/8 of the sleep outcomes compared to self-monitoring group. |
| | | | | | EMG Biofeedback vs. Sham | | Positive[A] | N/A | Sig. improvement in 5/8 of the sleep outcomes compared to pseudofeedback. |
| | | | | | EMG Biofeedback vs. WL | | Positive[NA] | N/A | Sig. improvement in 3/5 of the sleep outcomes reported compared to WL. |

| | | | | | | | | | |
|---|---|---|---|---|---|---|---|---|---|
| Yilmaz 2010 | 39 | 55.57 ± 7.17 [45-70]; 88% | Osteoarthritis of the knee | 2<br>N | EMG Biofeedback with PE vs. PE | Nottingham Health Profile: Sleep subscale | Mixed[A] | N/A | Sig. improvement in outcome over time with treatment (p<0.05) and a post-intervention difference between groups (p<0.02). No between group x time comparison given. |
| GUIDED IMAGERY | | | | | | | | | |
| Casida 2013 | 40 | 60.7 ± 15.6; 38% | Post-operative cardiopulmonary bypass surgery | 1<br>N | Guided Imagery vs. UC | VAS, AG | Negative[NA] | N/A | No sig. difference between group difference or overall time effect. |
| Renzi 2000 | 86 | 48 [25-72]; 51% | Anorectal disease surgery | 2<br>N | Guided Imagery vs. UC | SRS | Mixed[NA] | N/A | Sig. difference between intervention and control at endpoint (p=0.01). Between group over time comparisons not given. |
| Woolfolk 1983* | 44 | 43.3 [19-71]; 68% | Insomnia | 1<br>Y | Guided Imagery vs. WL | SD | Negative[NA] | Positive[NA] | Sig. improvement in only 1/6 sleep outcomes compared to control. Sig improvement in 5/6 sleep outcomes compared to control at 24 weeks follow-up |
| | | | | | Guided Imagery + Muscle tension-release vs. WL | | Negative[NA] | Positive[NA] | No sig. differences in sleep outcomes post-intervention. Sig improvement in 5/6 sleep outcomes compared to control at 24 weeks follow-up |
| HYPNOTHERAPY | | | | | | | | | |
| Abrahamsen 2008 | 41 | 56 ± 12.2; 85% | Orofacial, persistent idiopathic pain | 2<br>N | Hypnotherapy + PMR + Guided Imagery vs. PMR + Guided Imagery | PSQI | Negative[A] | N/A | Pre-post improvement in sleep quality in both groups but no sig. between-group differences (p < 0.11) |
| Abrahamsen 2009 | 40 | 38.6 ± 10.8; 100% | Temporo-mandibular disorders | 2<br>N | Hypnotherapy + PMR + Guided Imagery vs. PMR + Guided Imagery | PSQI | Negative[A] | N/A | Sig. pre-post reduction (p < 0.006) and control groups on NWAK - Pain; no other significant effects of time or group, but tendency of greater improvement of other sleep outcomes in HT group. |

| | | | | | | | | | |
|---|---|---|---|---|---|---|---|---|---|
| Abramowitz 2008 | 32 | 31.7; 0% | PTSD with insomnia | 2 Y | Hypnotherapy vs. Zolpidem | VSSQQ | Positive[A] | Mixed[A] | Quality of sleep improved with HT ($p=0.003$) and improved compared to control ($p<0.0005$). Improvement in number of awakenings and TST. |
| Barabasz 1976 | NR | NR | Mild depressive neurosis | 1 Y | Hypnotherapy + Cerebral Electrotherapy, vs. Cerebral Electrotherapy | SRS | Positive[A] | N/A | Both groups that isolated the HT effect significantly improved self-reported sleep ($p<0.05$). |
| Castel 2012 | 93 | 50.2 ± 6.2; 97% | Fibromyalgia | 2 N | Hypnotherapy + CBT vs. CBT | MOS-SS | Negative[A] | Negative[A] | Sig. improvement in sleep scores for CBT plus hypnotherapy and CBT alone ($p<0.001$). No sig difference between these two. |
| Elkins 2008 | 51 | 58; 100% | Breast cancer survivors with hot flashes | 2 N | Hypnotherapy vs. NTC | MOS-SS | Positive[NA] | N/A | Sig. improvement ($p<0.001$) in sleep with HT versus the control group. |
| Elkins 2013 | 174 | 54.6; 100% | Postmenopausal with hot flashes | 2 N | Hypnotherapy vs. Structured attention control | PSQI | Positive[A] | Positive[A] | Sig. improvement both after intervention ($p<0.001$) and at 12 week follow-up. |
| Farrell-Carnahan 2010 | 38 | 56.7 ± 11.7; 86% | Cancer survivors with insomnia | 1 Y | Hypnotherapy, self vs. WL | ISI, SRS | Negative[NA] | N/A | No sig. difference between groups. |
| Picard 2013 | 59 | 481. ± 9.3; 100% | Fibromyalgia | 2 N | Hypnotherapy vs. WL | MOS-SS | Negative[NA] | Positive[NA] | No sig. difference between groups following treatment ($p>0.05$), but sig improvement seen at 6 months ($p<0.01$). |
| Stanton 1989 | 45 | [23-67; 58%] | Insomnia | 1 Y | Hypnotherapy vs. Stimulus control | SRS | Positive[A] | N/A | Sig. improvement in SOL with HT compared to stimulus control ($p<0.05$). |
| | | | | | Hypnotherapy vs. Sham Hypnotherapy | | Positive[A] | N/A | Sig. improvement in SOL with HT compared to sham HT ($p<0.05$). |

| | | | | | | | | | |
|---|---|---|---|---|---|---|---|---|---|
| Whitehourse 1996 | 35 | 24.8; 60% | Medical students | 2 N | Hypnotherapy, self vs. WL | SD | Negative[NA] | N/A | Trend towards improvement in quality (p=0.06). Not all SD outcomes reported. |
| MEDITATION: MINDFULNESS | | | | | | | | | |
| Andersen 2013 | 247 | 54.2 ± 10.3; 100% | Post-surgery breast cancer | 1 N | Mindfulness Based Stress Reduction (MBSR) vs. UC | MOS-SS | Mixed[NA] | Negative[NA] | 3/9 subscale values sig diff from control at endpoint, although change scores for same time not sig. No sig. diff between groups in mixed model over time or at 6 and 12 month follow-ups |
| Britton 2010 | 21 | 47.7 ± 7.6; 77% | Depression - remitted | 1 Y | Mindfulness-Based Cognitive Therapy (MBCT) vs. WL | PSG, SD | Negative[NA] | N/A | No sig. differences in most of the outcomes measured by PSG and by SD. |
| Britton 2012 | 24 | 47.7 ± 8.0; 79% | Depression - antidepressant user | 1 Y | Mindfulness-Based Cognitive Therapy (MBCT) vs. WL | PSG, SD | Negative[NA] | N/A | No sig. difference in most PSG measurements. SD showed improvement in SE and TWT compared to control (p<0.05) but not in other measures. |
| Carmody 2011 | 92 | 53 ± 4.9; 100% | Late peri- and post-menopause with hot flashes | 2 N | Mindfulness Based Stress Reduction (MBSR) vs. WL | WHIIRS | Positive[NA] | Negative[NA] | Sig improvement compared to control (p=0.009). Intervention group score moved to below cutoff for problematic sleep disturbance. No sig difference at 20 wk follow-up |
| Esmer 2010 | 25 | 55.1 ± 10.3; 44% | Failed back surgery syndrome | 2 N | Mindfulness Based Stress Reduction (MBSR) vs. UC | PSQI-Abridged | Positive[NA] | Positive[NA] | Sig. improvement in sleep after the intervention (p = 0.047) and was maintained at 40 weeks. |
| Gross 2010 | 122 | 55 ± 11.3; 46% | Solid organ transplant recipients | 1 N | Mindfulness Based Stress Reduction (MBSR) vs. HE | PSQI | Positive[A] | Positive[A] | Sig. improvement in sleep between intervention and control (p=0.02) immediately and at 1 year follow-up (p<0.01). |
| Gross 2011 | 27 | 47 [21-65]; 75% | Insomnia | 1 Y | Mindfulness Based Stress Reduction (MBSR) vs. Eszopiclone | AG | Negative[A] | Negative[A] | No sig. difference between groups after intervention and no sig difference at 5 mth follow-up. |

| | | | | | | | | | |
|---|---|---|---|---|---|---|---|---|---|
| Klatt 2009 | 45 | 43.41 ± 2.17; 77% | Stressed working adults | 2 N | Mindfulness Based Stress Reduction (MBSR) vs. WL | PSQI | Mixed[NA] | N/A | Sig. pre-post improvement in 5/7 of the sleep measurements. No between group comparison was done. |
| Lengacher 2012 | 84 | 58 ± 9.4; 100% | Breast cancer | 2 N | Mindfulness Based Stress Reduction (MBSR) vs. WL | M.D. Anderson Symptom Inventory | Negative[NA] | N/A | No sig. difference between MBSR and control at 6 weeks (p=0.98). Improvement in both groups. |
| Malarkey 2013 | 170 | 51; 88% | University faculty & staff with risk for cardiovascular disease | 2 N | Low dose Mindfulness-Based Intervention vs. Lifestyle education | PSQI | Negative[A] | N/A | No sig. changes in PSQI global score. |
| Nakamura 2013 | 35 | 52.6 ± 9.8; 75% | Cancer survivor with sleep disturbance | 1 Y | Mindfulness; Mind-Body Bridging vs. SHE | MOS-SS | Mixed[A] | Positive[A] | Sig. improvement with MBB versus control immediately (p<0.001) and at 2 month follow-up (p=0.018). Near significance with MD versus control immediately (p=0.065). Sig improvement at 2 month follow-up (p=0.04). |
| Shapiro 2003 | 54 | 57 ± 9.7 [38-77]; 100% | Breast cancer | 1 N | Mindfulness Based Stress Reduction (MBSR) vs. Free choice control | SRS | Negative[A] | N/A | No sig. improvement between groups. |
| Stotter 2013 | 28 | 42.8 ± 12.6; 79% | Depression, moderate recurring or episodic | 2 N | Mindfulness-Based Touch Therapy & Mindfulness Counselling + UC vs. UC | HRSD-Sleep | Mixed[NA] | N/A | Sig. improvement in 1/3 outcomes compared to control at endpoint. No between group x time comparison given. |
| Wolever 2012* | 205 | 44.3 ± 9.4; 75% | Working adults | 2 N | MD - Mindfulness at Work vs. NTC | PSQI | Positive[NA] | N/A | Sig. decrease in sleep difficulties compared to control (p<0.05) |
| MEDITATION: OTHER | | | | | | | | | |

| | | | | | | | | | |
|---|---|---|---|---|---|---|---|---|---|
| Chan 2012 | 51 | 46.7 ± 8.1; 78% | Depression | 1 Y | Dejian Mind-Body Intervention vs. CBT | HRSD-Sleep & SD, SRS | Mixed[A] | N/A | Sig. pre-post improvement in the majority of measurements (p<0.05). No between group comparison done. |
| | | | | | Dejian Mind-Body Intervention vs. WL | | Mixed[NA] | N/A | |
| Milbury 2013 | 40 | 53.0 ± 6.6; 100% | Cancer, breast - post-chemotherapy w/ cognitive impairment | 2 N | Tibetan sound meditation vs. WL | PSQI | Negative[NA] | Negative[NA] | No sig. pre-post or between group effect on sleep. |
| Nakamura 2011 | 58 | 53.8 ± 10.4; 6% | Veterans with sleep disturbance | 1 Y | Mind-Body Bridging vs. SHE | MOS-SS | Positive[A] | N/A | Sig. improvement in overall score and 2/6 of the subscales compared to control (p<0.01). |
| Pinniger 2013 | 64 | 39.5 [18-68]; 89% | Stress, anxiety and/or depression, self-reported | 1 N | Clinically Standardized Meditation vs. WL | ISI | Negative[NA] | Negative[NA] | No sig. difference between groups. |
| Rybarczyk 1999 + Rybarczyk 2001 | 237 | 64.5 [50-87]; 82% | Chronic health condition; >50 years old | 2 N | Mind-body wellness vs. WL | MSCL - sleep difficulties | Positive[NA] | Positive[NA] | Sig. difference between groups immediately after intervention (p<0.05) and at 1 yr follow-up (p<0.05). |
| Schoicket 1988 | 65 | 52.14 ± 13.14 [28-75]; 77% | Insomnia | 1 Y | Meditation vs. SHE | SD | Negative[A] | N/A | No sig. difference between groups. All groups saw an improvement in sleep over time (p<0.05) |
| | | | | | Meditation vs. Stimulus control | | Negative[A] | N/A | |
| Wiriyasombat 2011 | 77 | 69.77 ± 6.46; 78% | Elderly | 1 N | Vipassana meditation vs. NTC | SRS - Thai Elderly Spritual Well-Being Study | Negative[NA] | Positive[NA] | No sig. change at post-intervention. Sig improvement at 4 month follow-up in intervention (<0.001). |
| MEDITATION: TM | | | | | | | | | |

| | | | | | | | | | |
|---|---|---|---|---|---|---|---|---|---|
| Brooks 1985 | 18 | 33.3; 0% | Veterans, post-Vietnam | 2 N | Transcendental meditation vs. Psychotherapy | SRS | Positive[A] | N/A | Sig. improvement in insomnia with intervention compared to control (p<0.01). |
| Travis 2009 | 38 | 22.4 ± 8.0; 74% | College students | 2 N | Transcendental meditation vs. WL | ESS | Positive[NA] | N/A | Sig. improvement in sleep after intervention compared to control (p=0.001). |
| MOVEMENT: YOGA | | | | | | | | | |
| Afonso 2012 | 44 | [50 - 65]; 100% | Post-menopause with insomnia | 1 Y | Yoga vs. Passive stretching | ISI | Negative[A] | N/A | No sig. difference between intervention and active control |
| | | | | | Yoga vs. WL | | Positive[NA] | N/A | Yoga presented significantly lower ISI when compared to WL (p<0.05). |
| Bower 2012 | 31 | 53.9 ± 5.3; 100% | Breast cancer survivor with fatigue | 2 N | Iyengar yoga vs. HE | PSQI | Negative[A] | Negative[A] | No sig. difference between groups in sleep disturbance. |
| Carson 2009 | 29 | 54.4 ± 7.5; 100% | Breast cancer survivor with hot flashes | 2 N | Yoga vs. NTC | SRS | Positive[NA] | Negative[NA] | Sig. improvement in sleep disturbance between intervention and control (p=0.0071). No sig at 1 month follow up |
| Chandwani 2010 | 56 | 52.7 ± 9.1; 100% | Breast cancer undergoing radiation | 2 N | Yoga vs. WL | PSQI | Negative[NA] | Negative[NA] | No sig. differences in sleep disturbance over time and between groups. |
| Chattha 2008 | 108 | 48.5 ± 3.8; 100% | Peri-menopause | 2 N | Integrated yoga therapy vs. PE | SRS - Vasomotor Symptom Checklist | Negative[A] | N/A | Nearing sig. difference between groups over time (p=0.08). |
| Chen KM 2009 | 128 | 69.2 ± 6.2; 84% | Elderly - community dwelling | 1 N | Silver yoga vs. NTC | PSQI | Positive[NA] | N/A | Sig. improvement in PSQI score for intervention compared to control (p<0.001). |
| Chen KM 2010 | 55 | 75.4 ± 6.70; 53% | Transitional frail elders | 1 N | Silver yoga vs. NTC | PSQI | Positive[NA] | N/A | Sig. improvement in PSQI score for intervention compared to control (p<0.005). |

| | | | | | | | | | |
|---|---|---|---|---|---|---|---|---|---|
| Cohen L 2004 | 38 | 51; 31% | Lymphoma | 1 N | Tibetan yoga vs. WL | PSQI | Positive[NA] | N/A | Sig. improvement after intervention in total PSQI score between intervention and control (p<0.004). 4/7 subscales of PSQI were sig. improved compared to control. |
| Dhruva 2012 | 16 | 54.2 ± 13.0; 88% | Cancer, undergoing chemotherapy | 2 N | Pranayama yoga vs. WL | GSDS | Mixed[NA] | N/A | Sig. improvement in sleep disturbance in yoga group (p<0.04). No between group comparison given. |
| Elavsky 2007 | 150 | 49.9 ± 3.6; 100% | Menopause | 1 Y | Iyengar yoga vs. PE | PSQI | Negative[A] | N/A | No sig. differences in sleep outcomes between walking, yoga, and no intervention. |
| | | | | | Iyengar yoga vs. NTC | | Negative[NA] | N/A | |
| Garfinkel 1998 | 42 | 48.9 ± 45; 76% | Carpal tunnel syndrome | 2 N | Iyengar yoga vs. UC | SRS | Negative[NA] | N/A | No sig. difference between groups with sleep disturbance. |
| Hariprasad 2013* | 87 | 75.3 ± 6.9; 60% | Elderly in nursing home | 1 N | Yoga vs. WL | PSQI | Positive[NA] | N/A | Sig. improvement in PSQI between intervention and control (p<0.001). |
| Innes 2012 | 20 | 58.4 ± 2; 100% | Post-menopausal (overweight, physically inactive) | 1 N | Iyengar yoga vs. Education film | PSQI, SRS | Positive[A] | N/A | Sig. improvement in 4/7 of the PSQI subscale measurements between intervention and control (p<0.05). |
| Kohn 2013 | 37 | 53 ± 12; 91% | Stress with related symptoms and diagnoses | 2 N | Yoga + UC vs. UC | ISI | Negative[NA] | N/A | No sig. difference regarding changes in ISI from baseline was noted between groups. |
| Manjunath 2005 | 55 | 71.5 ± 8.2; 70% | Elderly | 1 N | Yoga vs. WL | SRS | Negative[NA] | Negative[NA] | No sig. pre-post or between group effect on sleep. |
| Mustian 2013 | 321 | 54.1 ± NR; 96% | Cancer survivor | 1 Y | Yoga for Cancer Survivors + UC vs. UC | PSQI, AG | Positive[NA] | N/A | Sig. improvement in 6/11 measurements of sleep between intervention and control. |

| | | | | | | | | | |
|---|---|---|---|---|---|---|---|---|---|
| Sakuma 2012 | 82 | 32.6 ± 11.5; 100% | Child-care workers | 2 N | Yoga vs. NTC | GHQ-30 | Negative[NA] | N/A | No sig. difference between groups. |
| Vadiraja 2009 | 75 | NR; 100% | Breast cancer patients, stage 2 and 3 | 2 N | Yoga vs. supportive counseling | EORTC | Positive[A] | N/A | Sig. improvement yoga group compared to the control at the end of the trial (p=0.001) |
| Wolever 2012* | 205 | 44.3 ± 9.4; 75% | Working adults | 2 N | YG - Viniyoga Stress Reduction Program vs. NTC | PSQI | Positive[NA] | N/A | Sig. decrease in sleep difficulties compared to control (p<0.05) |
| Yurtkuran 2007 | 37 | 38 ± 14.2; 55% | Hemodialysis | 2 N | Yoga vs. NTC | VAS | Positive[NA] | N/A | Sig. improvement in sleep outcome with treatment compared to control (p = 0.04). |
| MOVEMENT: QI QONG | | | | | | | | | |
| Chen 2013 | 96 | 45.0 ± 8.1; 100% | Breast cancer undergoing radiotherapy | 2 N | Qigong vs. WL | PSQI | Negative[NA] | Negative[NA] | No sig. pre-post or between group effect on sleep. No sig at 1 month follow-up. |
| Liu 2012 | 12 | 55.6 [20-70]; 100% | Fibromyalgia | 2 N | Qigong vs. Sham Qigong exercise | PSQI | Mixed[A] | N/A | Borderline sig. improvement in sleep quality with the qi gong group compared to the sham qi gong group (p=0.058). |
| Lynch 2012 | 73 | 52.81; 94% | Fibromyalgia | 2 N | Qigong - Level 1 Chaoyi Fanhuan Qigong vs. WL | PSQI | Positive[NA] | Positive[NA] | Sig. imp in PSQI global vs WL immediately (p<0.001) and 2 mth (p<0.001), 4 mth (p =.003), and 6 mth (p=.008) follow-up. |
| MOVEMENT: RESSEGUIER | | | | | | | | | |
| Maddali Bongi 2010 | 41 | 45.5 ± 11.8; 93% | Fibromyalgia | 2 N | Rességuier method vs. WL | SRS | Mixed[NA] | Mixed[NA] | Sig. pre-post improvement on sleep at end of intervention (p < 0.001) and at end of 6 mth follow up (p < 0.01). Pre-post difference in control group not significant. No between group comparisons given. |
| MOVEMENT: TAI CHI | | | | | | | | | |

| | | | | | | | | | |
|---|---|---|---|---|---|---|---|---|---|
| Frye 2007 | 72 | 69.2 ± 9.26 [52-82]; 64% | Elderly | 2 N | Tai Chi vs. PE: LIE exercise | PSQI | Negative[A] | N/A | No sig. difference between the Tai Chi and the LIE exercise group. |
| | | | | | Tai Chi vs. NTC | | Positive[NA] | N/A | Sig. improvement with Tai Chi compared to non-active control (p<0.05). |
| Irwin 2008 | 112 | 59 – 86; 69% | Elderly - community-dwelling | 1 Y | Tai Chi vs. HE | PSQI | Positive[A] | N/A | Sig. improvement in Global and 4/7 sub scores compared to control. |
| Jones 2012 | 98 | 53.3; 93% | Fibromyalgia | 2 N | 8-form Yang style Tai Chi vs. HE | PSQI | Positive[A] | N/A | Sig. improvement on sleep quality with Tai chi compared to education control (P<0.0001). |
| Li 2004 | 118 | 75.4 ± 7.8; 82% | Elderly | 1 Y | 8-form Yang style Tai Chi vs. PE | PSQI, ESS | Positive[A] | N/A | Sig. improvement in overall PSQI score (p<0.001) and in 7/9 of the subscales compared to control. |
| Nguyen 2012 | 73 | 68.9 ± 5.1; 50% | Elderly - community-dwelling | 1 N | 24-form Tai Chi vs. NTC | PSQI | Positive[NA] | N/A | Sig. improvement in sleep with Tai Chi versus control (p < 0.001) |
| Wang WC 2010 | 29 | 76.53 ± 9.74; 69% | Cerebral vascular disorder, elderly | 2 N | Yang style Tai Chi vs. Rehabilitation | PSQI | Negative[A] | N/A | No sig. improvement compared to control. |
| Yeh 2008 | 18 | 64.2 ± 16.2; 50% | Chronic heart failure | 1 N | Tai Chi + UC vs. UC | Sleep spectrogram technique | Positive[NA] | N/A | Sig. improvement in outcome compared to control (p<0.05). |
| MULTIPLE MBI | | | | | | | | | |
| Carlson 2001 | 44 | 34.6; 77% | Myofascial pain | 2 N | Physical self-regulation: Breathing, postural relaxation, proprioceptive retraining vs. UC | PSQI | Mixed[NA] | Negative[NA] | Both intervention and control improved after treatment. No sig. difference at 26 weeks follow-up. |

| | | | | | | | | | |
|---|---|---|---|---|---|---|---|---|---|
| Chen YL 2010 | 15 | 38.64 ± 3.7; 68% | Chronic pain | 2 N | PMR + Guided imagery vs. WL | SRS | Mixed[NA] | N/A | 75% improvement in sleep quality compared to 28.57% in control group, but no statistics reported |
| Cohen 2007 | 114 | 51.8 ± 11.6 [27-74]; 100% | Breast cancer | 2 N | Relaxation + Guided imagery vs. CBT | SRS | Positive[A] | Positive[A] | Sig. improvement in sleep difficulties after intervention (p<0.001) and at 6 month follow-up (p<0.01). |
| | | | | | Relaxation + Guided imagery vs. NTC | | Positive[NA] | Positive[NA] | |
| Field 2013 | 75 | 26.6 ± 5.5; 100% | Prenatal depression | 2 N | Yoga + Tai Chi vs. WL | SRS VAS | Positive[NA] | N/A | Sig. improvement in sleep compared to control (p=0.05) |
| Richards 1998 | 70 | 65.8 [55-79]; 0% | CVD pts in critical care units, Elderly | 1 N | PMR + Guided imagery vs. UC | PSG | Negative[NA] | N/A | No sig. difference between groups. |
| | | | | | PMR + Guided imagery vs. Massage | | Negative[A] | N/A | |
| Richardson 2003 | 36 | 58.4 ± 14.3; 53% | Critically ill | 1 N | PMR + Relaxation Imagery vs. UC | VSH | Negative[NA] | N/A | No sig. difference between groups |
| Sendhikumar 2013 | 20 | 32.3 ± 9.911 [20-55]; 35% | Guillain-Barre syndrome | 2 N | Pranayama Yoga + Meditation + UC vs. UC | PSQI | Positive[NA] | N/A | Sig. improvement in treatment group compared to control (p=0.048). |
| Sumter 2009 | 33 | NR; 100% | Prison Inmates | 2 N | Mindfulness - breath, mantra, walking, yoga vs. NTC | SRS | Mixed[NA] | N/A | Sig. improvement in 1/2 outcomes compared to control at endpoint. No between group x time comparison given. |

| | | | | | | | | | |
|---|---|---|---|---|---|---|---|---|---|
| Sun 2013 | 75 | 69.7 ± 8.0; 75% | Elderly - community dwelling | 1 Y | PMR + meditation + SHE vs. SHE | PSQI, ESS | Positive[A] | N/A | Sig. improvement in sleep after intervention compared to control (p<0.001). |
| Toussaint 2012 | 21 | 48 [27-56]; 90% | Fibromyalgia | 2 N | Amygdala training + UC vs. UC | ESS | Negative[NA] | N/a | No sig. differences between groups. |
| Yang 2010 | 79 | 49.6 ± 10.8 [22-71]; 31% | Nasopharyngeal Carcinoma | 2 N | PMR + Guided imagery vs. Chinese calligraphy handwriting | SDS Modified | Positive[A] | N/A | Sig. improvement in insomnia compared to calligraphy. |
| | | | | | PMR + Guided imagery vs. NTC | | Positive[NA] | N/A | Sig. improvement in insomnia compared to NTC |
| RELAXATION: PMR | | | | | | | | | |
| Bae 2012 | 24 | 23.5 ± 8.6; 44% | Atopic dermatitis | 2 N | PMR + UC vs. UC | SRS (loss of sleep) | Mixed[NA] | N/A | Loss of sleep was sig. decreased with PMR (P = 0.007) (but not Control). Group x time data NR |
| Borkovec 1976 | 33 | NR | Sleep disturbance in college students | 1 Y | PMR vs. Placebo | PSG, SD | Mixed[A] | Positive[A] | Sig. improvement in sleep latency and PSG stage 1 sleep (p<0.05). No between group comparisons given. Sig. improvement in sleep latency at 1 year with intervention (p<0.01). |
| | | | | | PMR vs. WL | | Mixed[NA] | N/A | Sig. improvement within group for almost all PSG measurements (p<0.05). No between group comparisons given. |
| Cannici 1983 | 30 | 55.8 [21-80]; 63% | Cancer patients with insomnia | 1 Y | PMR vs. UC | SD | Negative[NA] | N/A | No sig. difference between groups in 8/9 of SD outcomes measured. |
| Ducloux 2013 | 18 | 66 ± 10.7; 67% | Metastatic cancer | 1 Y | PMR vs. WL | SRS | Negative[NA] | Negative[NA] | No sig. between group difference regarding post-intervention sleep satisfaction scores. |

| | | | | | | | | | |
|---|---|---|---|---|---|---|---|---|---|
| Edinger 2001 | 70 | 54.5 ± 10.2; 44% | Insomnia, persistent | 1 y | PMR vs. CBT and placebo desensitization | PSG, SD, SRS | Negative[A] | N/A | Active control sig. better than Mindbody group on objective measures. PMR was compared to both groups together in analysis and not separately to each group. |
| Engle-Friedman 1992 | NR | 60.26; NR | Insomnia, Elderly | 1 Y | PMR + Support + SHE vs. Support + SHE | PSG, SD | Negative[A] | NR | No sig. difference between the treatment and active controls. All showed some improvement. |
| | | | | | PMR + Support + SHE vs. Support + SHE + Stimulus control | | Negative[A] | NR | |
| | | | | | PMR + Support + SHE vs. NTC | | Positive[NA] | NR | Sig. improvement over inactive control (p<0.05) for most of the SD and PSG outcomes. |
| Espie 1989 | 70 | 48.1 ± 10.6; 50% | Insomnia | 1 Y | PMR vs. Stimulus control | SRS | Positive[A] | Negative[A] | Sig. improvement in all three of the active groups (p<0.001). PMR only intervention that showed improvement in both the rested measurement and sleep enjoyment measurement. No sig. improvement seen at 3, 6, and 17 mth follow-up. |
| | | | | | PMR vs. Paradoxical intention | | Positive[A] | Negative[A] | |
| | | | | | PMR vs. Placebo desensitization | | Positive[A] | Negative[A] | |
| Field 1999 | 26 | 29.5 ± 2.7; 100% | Pregnant | 2 N | PMR vs. Massage | SRS | Negative[A] | N/A | No sig. improvement in sleep with PMR. |
| Francis 2012 | 60 | NR; 50% | Admitted in hospital ward | 1 Y | PMR vs. NTC | PSQI | Mixed[NA] | N/A | Sig. improvement in some of the PSQI subscales (P<0.05). |
| Freedman 1976* | 18 | [17-39]; 56% | Insomnia | 1 Y | PMR vs Williams exercises | PSG | Negative[A] | Negative[A] | Sig. improvement in 1/5 of the PSG measurements reported at 2 weeks but not at 2 month follow-up. |

| | | | | | | | | | |
|---|---|---|---|---|---|---|---|---|---|
| Greeff 1998 | 22 | 45.5 ± 9.5; 0% | Chronic alcoholics with insomnia | 1<br>Y | PMR vs. NTC | SRS Tworetzky | Positive[NA] | N/A | Sig. improvement in sleep quality with intervention vs control (p<0.001). |
| Ireland 1985 | 28 | 55.9 [28-76]; 53% | Tinnitus | 2<br>N | PMR vs. WL | SD | Negative[NA] | N/A | No sig. change in sleep onset. |
| Lacks 1983 | 64 | 40.6; 75% | Insomnia | 1<br>Y | PMR vs. Stimulus control | SD | Negative[A] | N/A | Stimulus control sig. better than PMR |
| | | | | | PMR vs. Paradoxical intention | | Negative[A] | N/A | No sig. difference between PMR and paradoxical intention |
| | | | | | PMR vs. Placebo desensitization | | Negative[A] | N/A | No sig. difference between PMR and placebo desensitization |
| Lichstein 1999 | 30 | 52 [21-80]; 58% | Insomnia | 1<br>Y | PMR vs. NTC | SD | Positive[NA] | N/A | Sig. improvement in 2/5 of the sleep outcomes measured. |
| Lick 1977 | 40 | 47.48 ± 10.88; [29-72]; 65% | Insomnia | 1<br>Y | PMR; PMR+ tape vs. Placebo | SRS | Positive[A] | N/A | Sig. improvement in 2/4 of the sleep outcomes with intervention compared to placebo (p<0.05) |
| | | | | | PMR; PMR + tape vs. NTC | | Positive[NA] | N/A | Sig. improvement in most of the sleep outcomes with intervention compared to NTC (p<0.05) |
| Nicassio 1982* | 22 | 43.5 [17-64]; 78% | Insomnia | 1<br>Y | PMR vs. NTC | SRS, SD | Positive[NA] | N/A | Sig. improvement with PMR versus no treatment controls (p <0.05). |
| Wang WH 2012 | 130 | [30-68]; 18% | Obstructive sleep apnea | 2<br>Y | PMR vs. UC | ESS, PSQI | Negative[NA] | N/A | No sig improvement with PMR alone compared to control. |
| | | | | | PMR + HE vs. UC | | Positive[NA] | N/A | Sig. improvement with PMR+ education compared to control with both outcomes (p<0.05). |

| | | | | | | | | | |
|---|---|---|---|---|---|---|---|---|---|
| | | | | | PMR vs. HE | | Negative[A] | N/A | No sig improvement with PMR alone compared to HE. |
| | | | | | PMR + HE vs. HE | | Positive[A] | N/A | Sig. improvement with PMR + HE compared to education only with both outcomes (p<0.05). |
| Waters 2003 | 53 | 46.3; 70% | Insomnia | 1 Y | PMR vs. Medication | SD | Mixed[NA] | N/A | Sig. improvement in 5/6 outcomes over time. Between group comparison was not reported. |
| | | | | | PMR vs. sleep restriction and stimulus | | | | |
| | | | | | PMR vs. SHE | | | | |
| Wilson 1982 | 64 | 38.8 [20-55]; 66% | Depression | 2 N | PMR vs. Amitriptyline | SRS | Negative[A] | N/A | No sig. difference between groups. |
| | | | | | PMR vs. Placebo | | Negative[A] | N/A | No sig. difference between groups. |
| Woolfolk 1983* | 44 | 43.3 [19-71]; 68% | Insomnia | 1 Y | PMR vs. WL | SD | Negative[NA] | Negative[NA] | No sig. differences in sleep outcomes post-intervention or at 24 week follow-up |
| RELAXATION: OTHER | | | | | | | | | |
| Giblin 1983 | 20 | 70.4 [56-80]; 90% | Insomniacs taking hypnotic meds for 6+ months | 1 Y | Autogenic Relaxation vs. NTC | SRS | Negative[NA] | NFU | No sig. difference between groups. |
| Gustavsson 2006 | 33 | 43; 100% | Musculoskeletal neck pain | 2 N | Applied Relaxation vs. UC | SRS | Negative[NA] | Negative[NA] | No sig. difference between groups after intervention and no sig difference at 20 week follow-up. |

| | | | | | | | | | |
|---|---|---|---|---|---|---|---|---|---|
| Lindh-Astrand 2013 | 58 | 54.9 ± 5.5; 100% | Postmenopausal with hot flashes | 2 N | Applied Relaxation vs. NTC | Women's Health Questionnaire | Positive[NA] | Positive[NA] | Sig. better improvement in sleep-related quality of life scores both after intervention (p<0.05) and at 3 mth follow-up (p<0.01). |
| Rambod 2013 | 83 | 49.9 ± 12.5; 36% | Hemodialysis | 1 N | Benson vs. UC | PSQI | Mixed[NA] | N/A | Sig. improvement in 5/7 of the outcomes seen at the endpoints between groups (p<0.05). No between group over time comparisons given. |
| Rybarczyk 2002 | 41 | 67.8; 58% | Insomnia | 1 Y | Home-based audio relaxation treatment vs. CBT | SRS | Negative[A] | Negative[A] | Only 1/6 outcomes were sig. improved from control. No sig. at 4 mth follow-up. |
| | | | | | Home-based audio relaxation treatment vs. WL | | Positive[NA] | Positive[NA] | Sig. improvement in 2/6 outcomes compared to control (p<0.05) immediately after treatment and in 3/6 outcomes at 4 mth follow-up. |

AG: actigraphy; CBT: cognitive behavioral therapy; EEG: electroencephalography; EMG: electromyography; EORTC: European Organization for Research and Treatment of Cancer; ESS: Epworth Sleepiness Scale; GHQ: general health questionnaire; GI: guided imagery; GSDS: General Sleep Disturbance Scale; HE: health education; HRSD: Hamilton Psychiatric Rating Scale for Depression; HRV: Heart rate variability; HT: hypnotherapy; ISI: Insomnia Severity Index; MBCT: Mindfulness-Based Cognitive Therapy; MBSR: Mindfulness Based Stress Reduction; MOS-SS: Medical Outcomes Study Sleep Scale; NTC: No treatment control; PE: physical exercise; PMR: progressive muscle relaxation; PSG: Polysomnography; PSQI: Pittsburgh Sleep Quality Index; PTSD: posttraumatic stress disorder; SBQ: Sleep Behavior Questionnaire; SD: sleep diary; SDS: symptom distress scale; SHE: sleep hygiene education; SMR: sensorimotor rhythm; SRS: self-reported scale; UC: usual care; VAS: visual analog scale; VSSQQ: Visual Subjective Sleep Quality Questionnaire; WHIIRS: Women's Health Initiative Insomnia Rating Scale; WL: waitlist

*The studies were classified as positive if >25% of measured sleep items had p value <0.05 compared to control group, mixed if close to 25% of measured items were positive; OR both intervention and control group improved but were not significantly different from each other OR intervention improved and not control but no reported direct comparison), or negative if <25% of measured items had p value <0.05 compared to the control group).

A = active control comparison
NA = non-active control comparison
